# Spontaneous Surface Charging and Janus Nature of the Hexagonal Boron Nitride–Water Interface

**DOI:** 10.1021/jacs.5c07827

**Published:** 2025-08-06

**Authors:** Yongkang Wang, Haojian Luo, Xavier R. Advincula, Zhengpu Zhao, Ali Esfandiar, Da Wu, Kara D. Fong, Lei Gao, Arsh S. Hazrah, Takashi Taniguchi, Christoph Schran, Yuki Nagata, Lydéric Bocquet, Marie-Laure Bocquet, Ying Jiang, Angelos Michaelides, Mischa Bonn

**Affiliations:** † Department of Molecular Spectroscopy, 28308 Max Planck Institute for Polymer Research, Ackermannweg 10, Mainz 55128, Germany; ‡ Yusuf Hamied Department of Chemistry, 2152 University of Cambridge, Lensfield Road, Cambridge CB2 1EW, United Kingdom; § Cavendish Laboratory, Department of Physics, 2152 University of Cambridge, Cambridge CB3 0HE, United Kingdom; ∥ International Center for Quantum Materials, School of Physics, 12465 Peking University, Beijing, 100871, China; ⊥ Laboratoire de Physique de l’École Normale Supérieure, Université PSL, Paris 75005, France; # Lennard-Jones Centre, University of Cambridge, Trinity Ln, Cambridge CB2 1TN, United Kingdom; ∇ Research Center for Materials Nanoarchitectonics, National Institute for Materials Science, Tsukuba 305-0003, Japan; ¶ New Cornerstone Science Laboratory, Peking University, Beijing 100871, China

## Abstract

Boron, nitrogen, and carbon are neighbors in the periodic table and can form strikingly similar twin structures—hexagonal boron nitride (hBN) and graphene—yet nanofluidic experiments demonstrate drastically different water friction on them. We investigate this discrepancy by probing the interfacial water and atomic-scale properties of hBN using surface-specific vibrational spectroscopy, atomic-resolution atomic force microscopy (AFM), and machine learning-based molecular dynamics. Spectroscopy reveals that pristine hBN acquires significant negative charges upon contacting water at neutral pH, unlike hydrophobic graphene, leading to interfacial water alignment and stronger hydrogen bonding. AFM supports that this charging is not defect-induced. pH-dependent measurements suggest OH^–^ chemisorption and physisorption, which simulations validate as two nearly equally stable states undergoing dynamic exchange. These findings challenge the notion of hBN as chemically inert and hydrophobic, revealing its spontaneous surface charging and Janus nature, and providing molecular insights into its higher water friction compared to carbon surfaces.

## Graphical abstract

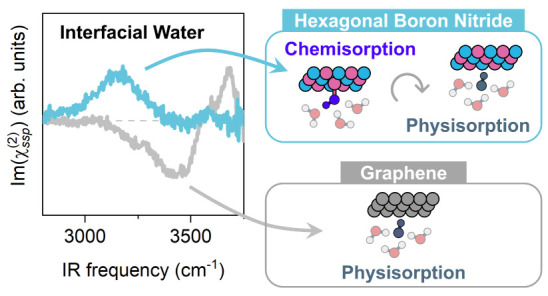

## Introduction

Water transport at the nanoscale plays a crucial role in numerous biological and industrial processes, from neurotransmission to ultrafiltration,^1,2^ thus attracting substantial interest. Recent advances in nanofluidics have enabled the development of artificial nanochannels with dimensions as small as a few angstroms, using atomically smooth surfaces like one-dimensional (1D) channels formed by carbon and boron nitride nanotubes,^3,4^ as well as two-dimensional (2D) channels made from 2D materials such as graphene and hexagonal boron nitride (hBN).^5–8^ These developments have facilitated a deeper exploration of water transport properties at the nanoscale, uncovering unexpected and significant differences in water’s hydrodynamic friction on those atomically smooth surfaces, with hBN consistently exhibiting one to 2 orders of magnitude higher friction than graphene, whether quantified by mass transfer,^6^ boundary slip length^4,9^ or friction coefficient.^7,10^ While current theories of solid–liquid interfaces typically describe the solid as a static external potential that influences the behavior of fluid molecules, with friction primarily attributed to the solid’s surface roughness,^11^ only a three to five times difference in water’s hydrodynamic friction is expected^12,13^ given that hBN and graphene share similar allotropic forms, which are often considered comparable in terms of surface roughness and presumed hydrophobicity.^14,15^ Indeed, several additional anomalous phenomena/properties of water have been observed in hBN nanoconfinement, such as spontaneous hydrolysis,^16^ osmotic energy conversion,^17^ atypical aqueous ion transport,^18^ and giant ferroelectric-like in-plane dielectric constant and notably enhanced in-plane conductivity.^19^

These observations point to an unexpectedly strong interaction of water with hBN, but the underlying mechanism has remained elusive or controversial. Previous studies have relied primarily on theoretical and computational simulations,^12–14,20–24^ while experimental insights remain scarce. Remarkably, nanofluidics experiments have indicated that surface charging for hBN in contact with water may serve as a possible explanation.^6,8,17,18,22,25,26^ While surface charges would indeed substantially enhance the interaction of hBN with water, the possible origin of the charge remains debated. For instance, while atomically flat 2D materials are typically considered charge-neutral and hydrophobic,^14,27^ theoretical studies have suggested that hydroxide (OH^–^) ions, a product of water autoionization, may exhibit an affinity for the hBN surface.^16,23,24^ This indicates that the hBN surface might undergo surface charging through the adsorption of OH^–^ when interacting with water. Such external surface charging could impact water transport by enhancing electrostatic interactions^24,28^ and by roughening locally the flat sheet. Furthermore, it has been proposed that defects on the hBN surface, often inevitable during crystal growth, may influence water transport similarly to the charging effect.^20–22^ Both mechanisms provide plausible explanations for the differences in water transport behavior between hBN and graphene and challenge the notion of hBN’s “chemical inertness.” Yet, while the defect scenario is extrinsic and could potentially be mitigated by treatment or using improved hBN, the adsorption of OH^–^ ions sets an intrinsic limitation on hBN’s properties for nanofluidics.

Clearly, molecular-level insights into the potential occurrence of surface charging at 2D materials (hBN and graphene)-water interfaces are essential to verify or falsify these mechanisms. Ideally, one would like access to the molecular-level details of the buried 2D material-water interface, including interfacial water structure, such as its orientation and hydrogen bond (H-bond) environment, as well as the hBN surface properties like defects and surface charges. Here, we provide such molecular-level insights by combining heterodyne-detected sum frequency generation (HD-SFG) spectroscopy, atomic-resolution atomic force microscopy (AFM), and machine learning-based molecular dynamics.

HD-SFG spectroscopy is an ideal tool for investigating the interfacial water structure and the potential presence of surface charges on 2D materials. As a surface-specific vibrational spectroscopy technique, HD-SFG selectively probes the water molecules at the interface,^14,29,30^ naturally excluding signals from bulk water due to the SFG selection rules.^31,32^ This method provides access to the complex *χ* ^(2)^ spectrum, where the imaginary part (Im­(*χ* ^(2)^)) reveals crucial information about the H-bond network and the absolute orientation of interfacial water molecules.^33,34^ Moreover, the additive nature of the *χ* ^(2)^ signals allows for the separation of contributions from water aligned as the result of surface charge, enabling the direct quantification of surface charges.^35,36^ These capabilities make HD-SFG spectroscopy particularly well-suited for probing interfacial water and surface charge information on the hBN, offering new experimental insights into its “chemical inertness.”

In addition to HD-SFG spectroscopy to examine the interfacial water structure and surface charges on hBN, we also use AFM to visualize surface defects in real space. Our method involves the preparation of high-quality, single-crystal hBN via mechanical exfoliation, yielding a defect-free hBN surface. The qPlus-based AFM measurements confirm the absence of defects, while the HD-SFG spectroscopy reveals that the interfacial water molecules form strong hydrogen bonds and are aligned up toward the defect-free, negatively charged hBN. Interestingly, the defect-free hBN surface exhibits significant negative charging when in contact with water, even at neutral pH, unlike graphene, which we show to remain charge-neutral and hydrophobic under similar conditions. We attribute this surface charging to the adsorption of OH^–^ ions on the hBN surface, supported by pH-dependent surface charge measurements from HD-SFG spectroscopy. Additionally, through machine learning-based molecular dynamics simulations with first-principles accuracy, we demonstrate that OH^–^ adsorption occurs in two almost equally stable states—chemisorbed and physisorbed. These states are also separated by a low energy barrier, facilitating dynamic interconversion between them. This behavior reflects spontaneous surface charging driven by dynamic interplay between physical and chemical adsorption—or the “Janus nature”—of the hBN surface when in contact with water. Our experimental results and atomistic simulations challenge the traditional view of hBN as “chemically inert” and offer new insights into the mechanisms behind surface charging in two-dimensional materials.

## Results and Discussion

We prepared a large-area (>200 × 200 μm^2^) hBN flake, approximately 100 nm thick, on a SiO_2_ substrate using the well-established polymer and solvent-free mechanical exfoliation method following the procedures described in the refs. 6,7 The procedures are detailed in the Methods. After flake preparation, a flat and clean region approximately 150 × 150 μm^2^ in size was identified using an optical microscope, and a 100 nm thick gold film was used to mark the identified area and encapsulate/cover the edges of the hBN for the HD-SFG measurement (for more details, see Methods S1–S3).

We ensured the selected region was clean and atomically smooth, free of visible wrinkles, edges, and hydrocarbon contamination (see Note S1 for details). Additionally, we confirmed that the ∼ 100 nm thick hBN layer effectively screened any potential influence of the supporting substrate on the interfacial water at the supported hBN/water interface (see Notes S2 and Note S3 for details). A schematic of the sample composition and beam geometry of SFG measurement is shown in Figure 1a, and an optical image of the prepared hBN sample from the bottom view is shown in Figure 1b. In this HD-SFG configuration, the visible (ω_vis_), local oscillator (LO), and infrared (ω_IR_) lights impinge noncollinearly from the optically transparent SiO_2_ substrate, passing through the SiO_2_ and the hBN flake, to overlap at the hBN/water interface. The reflected LO light interferes with the sum-frequency (ω_SFG_) signals generated from the water in the “reflected” direction, producing a heterodyned sum-frequency output that enables access to the complex *χ* ^(2)^ signals.

**Figure 1:**
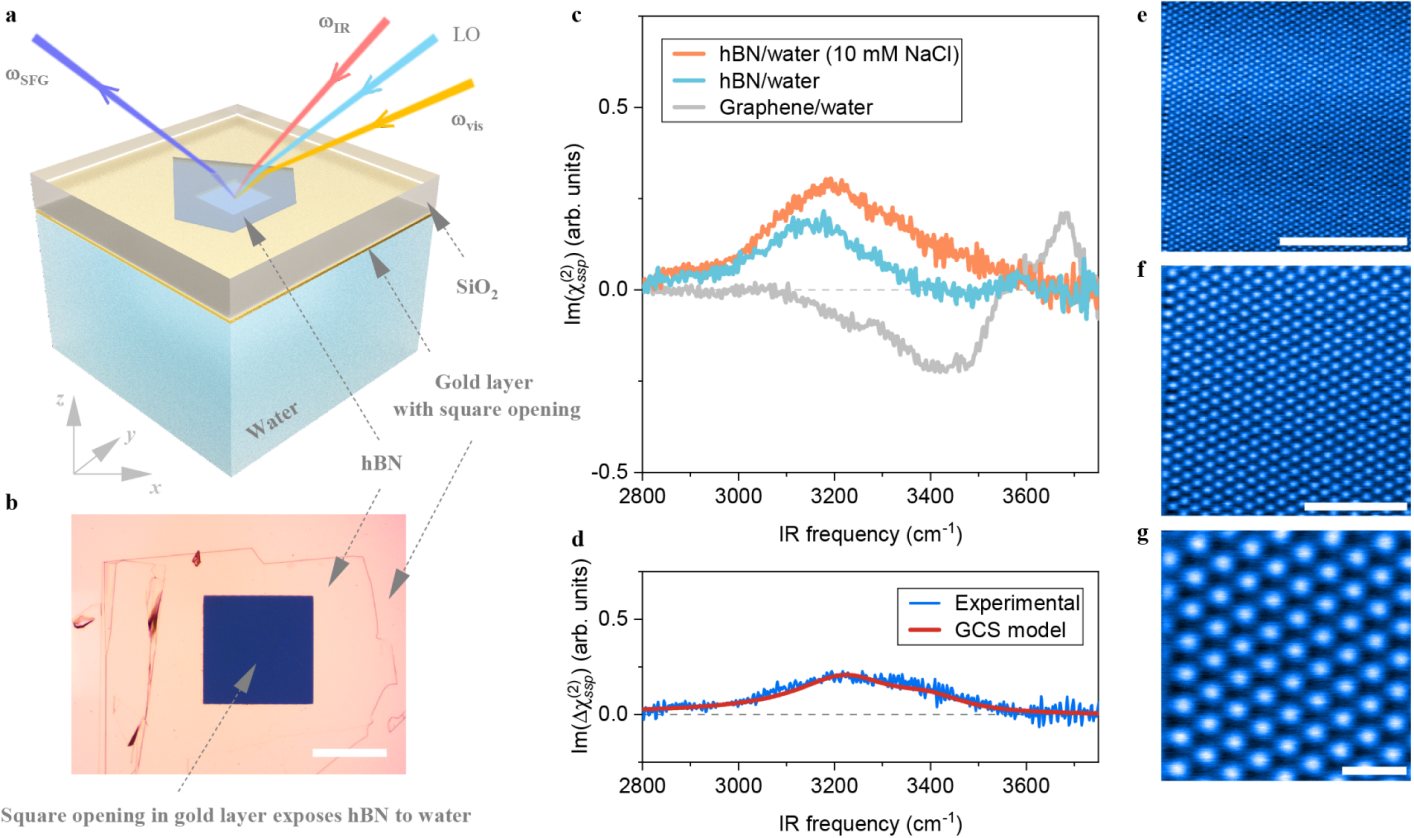
Interfacial water structure on hBN revealed by HD-SFG spectroscopy. a. A schematic of the composition of the hBN sample and beam geometry of the SFG setup. The hBN flake is positioned on an SiO_2_ substrate, with a gold layer encapsulating its edges. A square opening in the gold layer, located near the center of the hBN, exposes a portion of the hBN surface to water. The laser beams reach the hBN/water interface through the substrate. b. An optical image of the prepared hBN sample on a SiO_2_ substrate, encapsulated by gold along its edges, showing the bottom view of the setup described in (a). The scale bar corresponds to 100 μm. c. Experimental Im(χBN(2)) spectrum obtained for water and 10 mM NaCl at pH ∼ 6. Experimental Im(χG(2)) spectrum of the graphene/water interface is shown, with its amplitude magnified by a factor of 13 for visual comparison, including differences such as those arising from the Fresnel factor. d. Experimental difference spectrum Im(ΔχBN(2)) between 10 mM and 100 mM NaCl solutions, compared with a calculated spectrum based on the Gouy–Chapman-Stern (GCS) theory. The gray dashed lines in (c) and (d) represent zero lines. e. Constant-height AFM image of the hBN surface. f and g. Zoomed-in AFM images from (e), with B and N atoms depicted in white and black, respectively. The scale bars indicate 5, 2, and 0.5 nm, respectively. SFG, sum-frequency generation light; vis, visible light; IR, infrared light; ω , angular frequency of light; LO, local oscillator; arb. units, arbitrary units.

We conducted the HD-SFG measurement within the marked region on the hBN sample using our homemade flow cell at the *ssp* polarization combination with the three letters indicating the polarizations of the SFG, visible, and infrared light fields, respectively (Figure 1a, see Methods S4 for more details). The Im(χBN(2)) spectrum measured in the 2800–3750 cm^–1^ frequency region using pure water (pH ∼ 6) is displayed in Figure 1c. This spectrum exhibits primarily a broad positive O–H stretch peak spanning from 2900 cm^–1^ to 3500 cm^–1^. The positive sign of the peak indicates the O–H group of the interfacial water pointing up^37^ toward the hBN surface, and its low peak frequencies indicate that the O–H group forms strong H-bonds.^14,38^ The Im(χBN(2)) spectrum contrasts sharply with the Im(χG(2)) spectrum measured at the graphene/water interface (see Methods S5 for more experimental details), as shown in Figure 1c. The Im(χG(2)) spectrum closely resembles that of a hydrophobic interface, such as the air/water interface^31,32,39^ and alkane/water interface,^40–42^ featuring a broad negative hydrogen-bonded (H-bonded) O–H peak around 3400 cm^– 1^ and a positive high-frequency dangling O–H peak above 3600 cm^– 1^, originating from OH groups pointing up toward graphene.^14,27,43,44^ This suggests that the graphene surface is hydrophobic and chemically inert in contact with water, consistent with previous experimental measurements^27,45^ and theoretical predictions.^14,43^ Interestingly, earlier theoretical studies employing *ab initio* molecular dynamics (AIMD) predicted that the pristine hBN surface would likewise be hydrophobic, with an Im(χBN(2)) spectrum similar to Im(χG(2)) , showing a broad negative H-bonded O–H peak around 3400 cm^– 1^ and a positive high-frequency dangling O–H peak above 3600 cm^– 1 14^. However, our experimental Im(χBN(2)) spectrum reveals only a positively signed H-bonded O–H peak at a low frequency (∼3150 cm^– 1^), with no noticeable signature of the dangling O–H peak. Our finding implies that the hBN surface is not hydrophobic but hydrophilic and negatively charged when in contact with water at neutral pH. Notably, the absence of C–H peaks (2850–2950 cm^–1^) in these Im(χBN(2)) spectra underscores the cleanliness of the samples, free of hydrocarbon contamination.^46,47^ We also checked that the observed spectrum features do not arise from carbonate in the water (see Note S4 for more details).

To further support that the hBN surface is negatively charged upon contacting water, we measured the Im(χBN(2)) spectrum upon adding 10 mM NaCl to the water. At a charged interface, in addition to the surface contribution ( $\chi_{\mathrm{SL}}^{\left(2\right)}$ -term) arising mainly from the alignment of the topmost 1–2 layers of water (Stern layer, SL, often also referred to as the compact layer, CL, or bonded interfacial layer, BIL), the penetration of the electrostatic field into the bulk solution induces alignment and polarization of water molecules in the diffuse layer (DL), providing a bulk contribution ( $\chi_{\mathrm{DL}}^{\left(2\right)}$ -term) to the SFG signals. Within the Gouy–Chapman-Stern (GCS) electric double-layer (EDL) model,^36,48–54^ the total SFG response can be described as $\chi^{\left(2\right)}=\chi_{\mathrm{SL}}^{\left(2\right)}+\chi_{\mathrm{DL}}^{\left(2\right)}$ , where $\chi_{\mathrm{DL}}^{\left(2\right)}\left(c\right)\approx \chi^{\left(3\right)}\phi_{s}\left(c\right)\kappa \left(c\right)/\left(\kappa \left(c\right)-i\Delta k_{z}\right)$ . Here, χ^(3)^ primarily represents the third-order nonlinear susceptibility originating from bulk water, *ϕ*_s_ is the interfacial electrostatic potential at the plane (z_s_) that separates surface and bulk contributions, κ is the inverse of Debye screening length, and Δ*k*_z_ is the phase-mismatch of the sum-frequency, visible, and infrared beams in the depth (z) direction (see Note S5 for more discussion). The addition of electrolyte with concentration *c* screens the surface charge, thereby modulating both *ϕ*_s_ and κ which in turn alters the bulk contribution to the SFG signal.^55,56^ While the surface contribution remains only weakly affected,^36^ the bulk contribution, if present, should be significantly modulated—initially increasing with ion concentration, reaching a maximum around ∼ 1 mM, and then decreasing at higher concentrations due to optical interference effects and charge screening.^55,56^ The data, shown in Figure 1c, reveals a substantial modification of the water response, indicating the hBN surface is indeed charged. A quantitative analysis of the differential SFG signals at different ion strengths, confirms a significant bulk χ^(3)^ contribution peaked at around 3250 cm^–1^, whose positive sign further confirms the surface’s negative charge (Figure 1d). Following previous protocols within the GCS model,^36,48–51^ we infer from the differential SFG signal that the effective surface charge (σ_s_) at the hBN/water interface is −15 ± 6 mC/m^2^ at pH ∼ 6. Notably, within the GCS model, σ_s_ refers to the effective surface charge at the z_s_ plane, the exact location of which remains unclear.^36^ Nevertheless, it is generally agreed that σ_s_ is composed of the surface charge of the material and counter charge bound to the material^36,50–54^ (see Note S5 for more extensive discussion on the estimation of σ_s_).

What is the mechanism behind the surface charging of the hBN? The hBN surface may acquire negative charges upon contact with pure water mainly for two possible reasons: (i) the presence of defects such as boron vacancies,^20,25^ and (ii) the adsorption of OH^–^ ions,^24^ a product of water autoionization $\left(H_{2}O↔{\mathrm{OH}}^{-}+H^{+}\right)$ , on the hBN surface. To examine (i) the potential presence of defects on the hBN surface, we conducted qPlus-based AFM measurements. All AFM data were acquired at 6 K under ultrahigh vacuum conditions (<5 × 10^–10^ Torr) to probe potential atomic defects. The constant-height, high-resolution AFM images of the hBN surface from a randomly selected region, shown in Figure 1e-g, reveal a clean surface with a perfect hexagonal honeycomb structure without defects over an area of 100 nm^2^. We conducted the qPlus-based AFM measurements at five different randomly selected 100 nm^2^ regions and all data show the absence of defects on the hBN surface (see Note S6 for more AFM results). The estimated σ_s_ at the hBN/water interface is −15 mC/m^2^, corresponding to one charge per ∼ 11 nm^2^. The probability of not finding a defect at this charge density across five different areas of 100 nm^2^ is below ∼ 5 × 10^–21^, assuming Poisson distribution of defects (see Note S6 for details). We also confirm that neither water contact nor the SFG measurement induces observable defects on the initially defect-free hBN surface (see Note S6 for Raman data). We therefore conclude that defects are not the primary cause of the surface charging observed on the hBN surface.

The above analysis indicates that defects are not responsible and implies that the adsorption of OH^–^ ions on the hBN surface might be responsible for the negative surface charge. The hypothesis of adsorption of OH^–^ ions on the hBN surface is plausible, given the appearance of the positive peak with a low peak frequency at approximately 3150 cm^–1^ in the Im(χBN(2)) spectrum (Figure 1c). The peak frequency of 3150 cm^–1^ is about 100 cm^–1^ red-shifted compared to the bulk $\chi^{\left(3\right)}$ contribution (Figure 1d), which peaks at around 3250 cm^–1^ regardless of salt solution or surface properties.^36,48–50^ This redshift can be accounted for by interfacial water O–H groups donating strong H-bonds to OH^–^ at the hBN interface. Remarkably, the 3150 cm^– 1^ peak exhibits a continuum extending below 2900 cm^–1^, testifying to the strong interaction of water O–H groups with OH^–^ species.^57^ These water O–H groups, on average, point *up* toward the adsorbed OH^–^ on the hBN surface, which explains its positive sign.

These experimental findings strongly indicate that OH^–^ ions adsorb at the hBN interface, influencing the orientation of interfacial water molecules. The absence of a strong chemisorbed O–H signature in the Im(χBN(2)) spectrum, which would feature a negative high-frequency peak around 3600–3670 cm^– 1^ such as observed on CaF_2_^58^, sapphire,^59^ and silica surfaces,^60^ indicates a more complex adsorption behavior on hBN, possibly (also) involving physisorption rather than purely strong covalent bonding through chemisorption. Given the unexpected surface charging and the distinct spectral features observed, a deeper understanding of the underlying adsorption mechanisms is needed.

Motivated by these experimental observations, we conducted machine learning-based molecular dynamics (MD) simulations with first-principles accuracy for liquid water films at hBN interfaces (see Methods). Specifically, we investigated where OH^–^ ions adsorb at the interface and how they interact with water through a series of constrained and free MD simulations. A key result of this analysis is shown in Figure 2a where we report the potential of mean force (PMF) of an OH^–^ ion as a function of its distance from hBN. These simulations reveal two stable adsorption states on hBN, illustrated in Figure 2b. The first is a well-defined chemisorbed state with the OH^–^ covalently bonded to a boron atom of the hBN layer at approximately 1.6 Å. The second, which we refer to as the physisorbed state, has the OH^–^ solvated within the first contact layer of water at around 3.4 Å from the surface. The stabilities of the two states are similar, with a free energy (relative to an OH^–^ in the interior of the water film) of 0.09 ± 0.02 eV for the chemisorbed state and −0.02 ± 0.01 eV for the physisorbed state. The presence of two states is consistent with a previous AIMD study.^24^ However, the behavior seen here on hBN is in stark contrast to graphene, where only a physisorbed state is observed at approximately 3.4 Å, as demonstrated by different groups,^61,62^ highlighting a key difference between the two materials.

**Figure 2:**
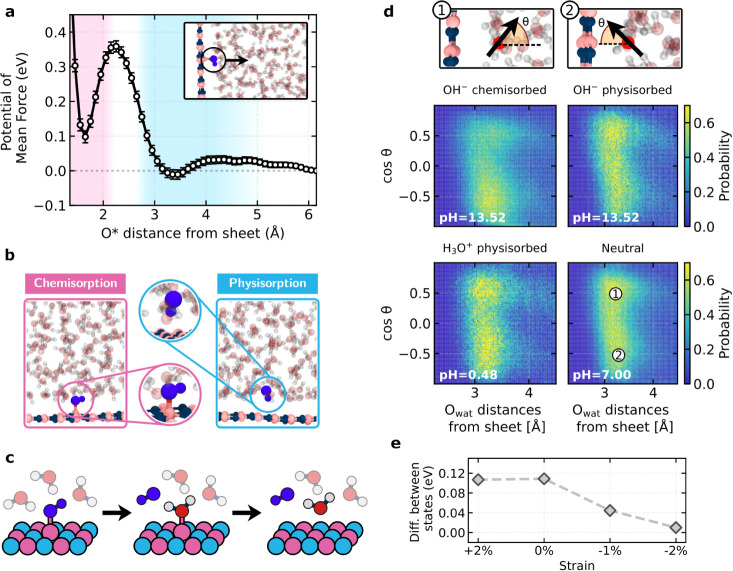
Surface chemistry of hBN revealed by machine learning-based MD simulations. a. Potential of mean force of an OH^–^ ion as a function of its oxygen distance from the hBN, obtained via umbrella sampling. b. Representative snapshots of the chemisorbed and physisorbed states, highlighting structural differences. c. Transition mechanism illustrating the protonation of chemisorbed OH^–^, followed by its desorption as a water molecule. d. Orientational distributions of interfacial water molecules under different pH conditions (basic, acidic, and neutral), showing distinct alignment patterns for chemisorbed and physisorbed OH^–^ ions. The angle definitions are shown in the accompanying schematics above. Dashed lines indicate the surface normal of the interfaces, while arrows represent the projection of the water molecule’s bisector onto the displayed plane. e. Free energy difference between the chemisorbed and physisorbed states as a function of strain applied to the hBN surface, indicating how mechanical strain influences the relative stability of these adsorption states. A positive value indicates that the physisorbed state is more stable.

Our free energy calculations show that the barrier between the chemisorbed and physisorbed states is low; approximately 0.2 eV to go from the chemisorbed to the physisorbed state. This low barrier, along with the similar free energy of the two states, suggests the possibility of dynamic exchanges between these two configurations, indicating a more intricate adsorption behavior than previously recognized.^24^ This finding points to an intriguing surface charging scenario involving both static (chemisorbed) and dynamic (physisorbed) surface charges. Indeed, upon running free MD, we see transitions from the chemisorbed to the physisorbed state on the nanosecond time scale, consistent with the barrier obtained from constrained MD (see Note S7). A closer inspection of the free MD simulations reveals an interesting transition mechanism: the chemisorbed OH^–^ first undergoes protonation before desorbing as a water molecule. This process is illustrated schematically in Figure 2c and is visible in Movie S1. Additionally, we examined the dynamics of the two states and found clear differences. In the chemisorbed state, OH^–^ remains relatively immobile, tightly bound to boron, while in the physisorbed state, it gains in-plane mobility, allowing freer diffusion along the surface (see Note S7). This distinction is particularly relevant for understanding nanoscale friction on hBN, as the mobility of surface-bound species can significantly influence interfacial slip and energy dissipation.

We now examine how the OH^–^ ion impacts the surrounding water in its chemisorbed and physisorbed states. Beyond their energetic similarities, these adsorption states exhibit distinct orientations along the surface normal, directly influencing the alignment of interfacial water molecules (see Figure 2d). Specifically, when the OH^–^ is in the physisorbed state the liquid water structure is similar to that of neutral water without any hydroxide. A similar effect is observed when H_3_O^+^ is present at the interface, where the surrounding water molecules retain their neutral water orientational distribution. In contrast, when the OH^–^ is chemisorbed, the hydrogen-bonded network of water is more structured with a peak in the orientational distribution at cos*θ*≈-0.5 corresponding to a preponderance of water molecules oriented toward the surface. This distinction was less evident in a previous AIMD study^14^ due to the limited time scales sampled (see Note S7 for further details). This again highlights the key role of machine learning-based MD simulations in enabling robust conclusions to be drawn from well-converged simulations.

Our machine learning based simulations reveal similar stabilities of the two states. Indeed, a 0.1 eV difference between the two states could easily be within the simulation error bar for a complex system such as this. For example, simulations of water are known to be sensitive to nuclear quantum effects and/or different exchange-correlation functionals.^63,64^ With this in mind, simulations reported in Note S7 show that these effects do slightly alter the relative stabilities of the two states. However, the key conclusion—that both states have a similar energy—is not altered. In addition, we show in Figure 2e that the relative stability of these adsorption states can be modulated by applying uniaxial strain to the hBN surface. This suggests an additional degree of control over OH^–^ adsorption, where external mechanical effects could shift the balance between chemisorption and physisorption. A compressive strain promotes a transition toward the chemisorption state, enhancing surface charging, whereas tensile strain favors physisorption. This observation is likely relevant to water confined in hBN nanotubes, where out-of-plane bending, strongly dependent on the nanotube radius, inevitably induces localized regions of both tensile and compressive strain.^65^

The complex interfacial chemistry at the hBN/water interface makes it challenging to quantitatively capture the thermodynamic driving forces behind OH^–^ chemisorption using AIMD simulations, particularly under neutral pH conditions, where the OH^–^ concentration is extremely low. To further show that OH^–^ adsorption on the hBN surface is thermodynamically favored, and to probe the resulting surface charging behavior, we measured the Im(χBN(2)) spectra while varying the OH^–^ ion concentration (pH). The ionic strength was maintained at 100 mM by adding NaCl to minimize bulk χ^(3)^ contributions, as shown in Figure 3a. The 3150 cm^– 1^ peak in the Im(χBN(2)) spectrum increases steadily as the pH increases from 4.5 to 11 but disappears at pH below 4.5. Simultaneously, the bulk contribution follows a similar trend with pH change: it is positive at pH values above 4.5 and negative below 4.5, with its intensity increasing at both higher and lower pH values. By comparing SFG spectra at different ion strengths,^36,48–50^ we infer that σ_s_ varies from +11 mC/m^2^ to −42 mC/m^2^ between pH = 3 and 11, reaching a minimum of approximately −0.5 mC/m^2^ at pH = 4.5 (Figure 3b). These results indicate that the isoelectric point of the hBN surface is around pH = 4.5, consistent with previous studies.^8,17,23^ Importantly, the consistent change of the 3150 cm^– 1^ peak and σ_s_ further supports our assignment of the 3150 cm^– 1^ peak to the O–H group of the topmost layer of water interacting with the adsorbed OH^–^ on the hBN surface. Notably, at pH 11, a weak yet discernible negative peak emerges at ∼ 3620 cm^–1^ (see Note S8 for additional data and discussion). The high frequency of this peak indicates a non-hydrogen-bonded O–H stretch, while its negative sign suggests that the O–H group is oriented down, toward the bulk solution. We thus assign this feature to the stretch vibrational mode of a chemisorbed OH group on the hBN surface. The appearance of this peak provides direct spectroscopic evidence that the negative surface charging of hBN originates from OH^–^ chemisorption.

**Figure 3:**
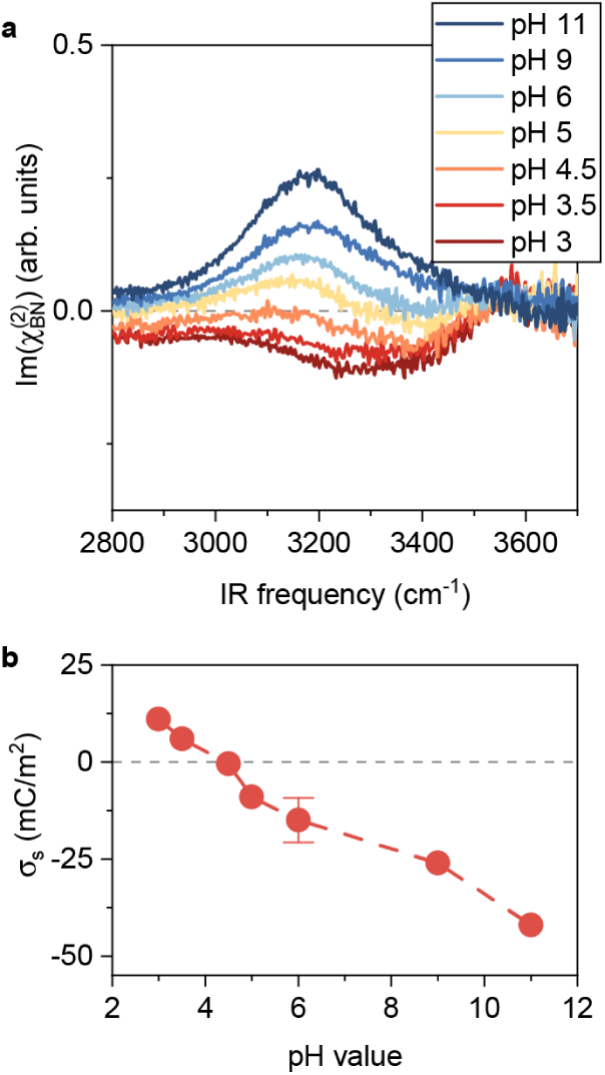
Surface chemistry of hBN revealed by HD-SFG spectroscopy. a. Experimental Im(χBN(2)) spectra obtained for 100 mM NaCl at various pH values. b. Inferred $\sigma_{s}$ from HD-SFG signals at various pH values. The gray dashed lines in (a) and (b) serve as zero lines.

Interestingly, at pH values below 4.5, the water at the interface responds like the hBN surface has become positively charged, and the Im(χBN(2)) spectra display a broad H-bonded O–H peak centered around 3350 cm^– 1^, as seen in Figure 3a. This can be accounted for by protons residing on the topmost layer, contributing to the positively charged interface. The surface propensity of protons has been previously confirmed both experimentally and theoretically for the air/water interface^57,66,67^ and the graphene/water interface,^61,68^ and is also consistent with our simulations shown in Figure 2d. Our experimental results suggest that the strong surface affinity of protons on hBN is already apparent at low proton concentrations (∼0.3 mM, pH = 3.5). We tentatively attribute this to the strong affinity of protons for the nitrogen atoms on the hBN surface, analogous to the strong affinity of hydroxide ions for the boron atoms. Regardless, the pH-dependent changes in the Im(χBN(2)) spectra provide compelling evidence that challenges the picture of hBN being “chemically inert” when in contact with water. Instead, these results reveal a strong affinity of both OH^–^ ions and protons for the hBN surface, giving rise to a negatively charged interface under mildly basic conditions and a positively charged interface under mildly acidic conditions. These results suggest that both hydroxide and hydronium adsorption must be considered to understand the surface charging behavior of the hBN/water interface. Although the underlying mechanisms, physical versus chemical adsorption, may differ, previous studies have emphasized the need to account for their competitive adsorption at the graphene/water interface.^62^ Taken together, these findings highlight that a comprehensive understanding of the surface charging behavior of 2D materials requires consideration of the competitive interactions of both hydroxide and hydronium ions with the surface.

## Conclusion

Our combined experimental and theoretical study challenges the traditional view of hBN’s “chemical inertness.” Contrary to conventional expectations of a hydrophobic surface, a defect-free hBN surface exhibits substantial negative charging when in contact with water at neutral pH, unlike graphene, which remains charge-neutral and hydrophobic. We provide experimental evidence that this surface charging in hBN arises from the adsorption of OH^–^ ions, a product of water autoionization, aligning with recent theoretical predictions.^23,24^ Remarkably, our experimental results suggest that the negative surface charge on hBN is already present under mildly acidic conditions (pH 4.5, OH^–^ concentration of ∼ 3 × 10^– 1 0^ M) and increases significantly as the pH rises and changes into positive at pH below 4.5. These findings offer molecular-level insights into surface charging mechanisms, prompting a reevaluation of hBN’s chemical behavior and intrinsic hydrophilicity. Using machine learning-based molecular dynamics simulations with first-principles accuracy, we further reveal that OH^–^ adsorption occurs in two states—chemisorbed and physisorbed—separated by a low energy barrier, allowing dynamic interconversion between them. Our experimental study was conducted using ∼ 100 nm-thick hBN flakes; however, the surface charging mechanism we reveal—arising from OH^–^ chemisorption—should be generalizable to thinner samples, including monolayer hBN. Since the chemisorption occurs at the surface layer, it is expected to be only weakly influenced by the underlying hBN layers. This is further supported by our AIMD simulations, which were performed for a monolayer hBN/water interface and successfully capture the key interfacial processes observed experimentally. This revised understanding may also explain the observed differences in water friction between carbon and hBN surfaces, highlighting the role of surface charging in these variations. Moreover, the inevitable pronounced surface charging due to OH^–^ ion adsorption on defect-free hBN in contact with water at neutral pH should be accounted for when discussing anomalous water properties near hBN surfaces or in nanoscale hBN confinement, such as spontaneous hydrolysis,^16^ osmotic energy conversion,^17^ atypical aqueous ions transport,^18^ and giant ferroelectric-like in-plane dielectric constant and notably enhanced in-plane conductivity.^19^

## Materials and Methods

### Sample Preparation

We employed high-quality hBN crystals for sample preparation, obtained from the International Center for Materials Nanoarchitectonics, National Institute for Materials Science 1–1 Namiki, Tsukuba 305–0044, Japan. hBN flakes were mechanically exfoliated using polydimethylsiloxane (PDMS) and dry-transferred onto an oxygen plasma-treated SiO_2_ substrate. This method ensures clean and large-area sample preparation. After preparation, a flat and clean region approximately 150 × 150 μm^2^ in size was identified using an optical microscope, and a gold structure was used to mark the identified area for the HD-SFG measurement. Notably, the thickness of the hBN flake was carefully chosen to be approximately 100 nm, ensuring that the SFG signal primarily probes the hBN/water interface, while minimizing contributions from the SiO_2_/hBN interface.^69^ The preparation of the suspended graphene on the water surface was similar to refs. 45,70 and was detailed in our recent work.^27^ More details of the sample preparation can be found in the Supporting Information.

### HD-SFG Measurement

HD-SFG measurements were performed on a noncollinear beam geometry with a Ti:sapphire regenerative amplifier laser system. A detailed description can be found in refs. 48,58 HD-SFG spectra were measured in a dried air atmosphere to avoid spectral distortion due to water vapor. To check the sample height, we used a height displacement sensor (CL-3000, Keyence). The IR, visible, and LO beams are directed at the sample (in SiO_2_) at incidence angles of approximately 34°, 43°, and 41°, respectively. We ensured the power of incident IR (∼3 μm) and visible (800 nm) pulses are far below the damage threshold value of a hBN crystal and do not introduce defects on the hBN surface (Note S9). The measurements were performed at the *ssp* polarization combination, where *ssp* denotes *s*-polarized SFG, *s*-polarized visible, and *p*-polarized IR beams. The HD-SFG signal at the hBN/water interface was normalized with that of the hBN/D_2_O interface. The suspended graphene sample HD-SFG spectra were normalized with that of the air/*z*-cut quartz. More details of the HD-SFG measurements can be found in the Supporting Information.

### qPlus-Based AFM Measurement

All experiments were conducted using a homemade system that combines scanning tunneling microscopy (STM) and noncontact atomic force microscopy (nc-AFM). The qPlus sensor was equipped with a tungsten (W) tip, characterized by a spring constant of approximately 1800 N·m^– 1^, a resonance frequency of about 28.9 kHz, and a quality factor of around 60000. All AFM data were collected at 6 K under ultrahigh vacuum conditions (<5 × 10^–10^ Torr). High-resolution AFM images were acquired in constant-height mode. A carbon monoxide (CO) tip was prepared on an Au(111) surface and subsequently transferred to hBN surfaces. Initially, a bare W tip was positioned directly above a CO molecule on the Au(111) surface (100 mV, 10 pA). The current was then increased to 500 pA, enabling the CO molecule to transfer to the tip apex. The oscillation amplitude of the qPlus sensor ranged from 100 to 500 pm. Image processing was performed using Nanotec WSxM software. The drift in tip–sample distance was minimal, with fluctuations of less than 1 pm over 8 min, and the temperature stability of our system improved to 0.01 K over 1 h. Fluctuations in amplitude and frequency shifts were limited to less than 4 pm and 30 mHz, respectively. These characteristics ensure stable, long-term high-resolution imaging.

### Machine Learning-Based Molecular Dynamics Simulations

All simulations were performed using a machine learning potential (MLP) based on the MACE architecture.^71^ We use 128 invariant channels, a cutoff distance of 6 Å, and two message-passing layers, resulting in an effective receptive field of 12 Å. The final energy and force root-mean-square errors of the model developed were 0.6 meV/atom and 19.4 meV/Å, respectively. The MLP developed and validated (see Methods S6) accurately represents the potential energy of the system and was trained using energies and forces obtained using the CP2K/Quickstep code.^72^ We specifically used the revPBE-D3 ^73,74^ functional as it accurately reproduces the structure and dynamics of liquid water^63,64,75^ and its ionized products.^76^ The Kohn–Sham orbitals of oxygen and hydrogen atoms are expanded using a TZV2P basis set, while those of boron and nitrogen are expanded using a DZVP basis set^77^ (see Methods S6), along with electronic structure settings consistent with previous work.^61^ The final model was trained on 8,402 structures encompassing the diverse range of conditions sampled, ensuring robust accuracy across different system configurations (see Methods). All MD simulations were performed at a temperature of 300 K in the NVT ensemble with a time step of 0.5 fs (see Methods S6). The systems (with no strain) were modeled using a 17.396 Å × 17.577 Å × 35.000 Å orthorhombic cell, containing 112 surface atoms, one OH^–^ ion, and 169 water molecules under periodic boundary conditions. To prevent interactions between periodic images, a 15 Å vacuum was included in the *z* direction, exceeding the model’s receptive field. In total, 5.05 ns of free MD and 3.96 ns of constrained MD simulations were performed, ensuring robust and statistically converged results. Constrained MD simulations were carried out using LAMMPS package^78^ and PLUMED,^79^ while free MD simulations were conducted using the ASE^80^ software.

## Supplementary Material





## Data Availability

All data needed to evaluate the conclusions in the paper are present in the paper and/or the Supporting Information.

## Notes

Experimental methods including sample preparation, HD-SFG measurements, simulation procedures, and model validation; supplementary notes on hBN surface cleanness, substrate effects, HD-SFG phase measurements, carbonate influence, surface charge analysis, hBN defect characterization, adsorption state stability, and laser fluence dependence­(PDF)
The chemisorbed OH^–^ first undergoes protonation before desorbing as a water molecule­(MP4)
