# Supplementary material for: Spontaneous Surface Charging and Janus Nature of the Hexagonal Boron Nitride–Water Interface
Source: J Am Chem Soc. 2025 Aug 6;147(33):30107–16. doi: 10.1021/jacs.5c07827 (PMC12371888; doi:10.1021/jacs.5c07827)
Supplement: Supplementary file 1 [file ja5c07827_si_001.pdf]

Supporting Information for

Spontaneous Surface Charging and Janus Nature of the Hexagonal Boron Nitride-Water Interface

Yongkang Wang[1†], Haojian Luo[1†], Xavier R. Advincula[2,3,4†], Zhengpu Zhao[5†], Ali Esfandiar[1,6], Da Wu[5], Kara D. Fong[2,4], Lei Gao[1], Arsh S. Hazrah[1], Takashi Taniguchi[7], Christoph Schran[3,4], Yuki Nagata[1], Lydéric Bocquet[6], Marie-Laure Bocquet[6], Ying Jiang[5,8], Angelos Michaelides[2,4], Mischa Bonn[1*]*

[1] Department of Molecular Spectroscopy, Max Planck Institute for Polymer Research, Ackermannweg 10, Mainz 55128, Germany.

[2] Yusuf Hamied Department of Chemistry, University of Cambridge, Lensfield Road, Cambridge CB2 1EW, United Kingdom.

[3] Cavendish Laboratory, Department of Physics, University of Cambridge, Cambridge CB3 0HE, United Kingdom.

[4] Lennard-Jones Centre, University of Cambridge, Trinity Ln, Cambridge CB2 1TN, United Kingdom.

[5] International Center for Quantum Materials, School of Physics, Peking University, Beijing 100871, China.

[6] Laboratoire de Physique de l'École Normale Supérieure, Université PSL, Paris 75005, France.

[7] Research Center for Materials Nanoarchitectonics, National Institute for Materials Science, Tsukuba 305-0003, Japan.

[8] New Cornerstone Science Laboratory, Peking University, Beijing 100871, China.

[*]Email: wangy3@mpip-mainz.mpg.de.

[*]Email: bonn@mpip-mainz.mpg.de.

[†]Y.W., H.L., X.R.A., and Z.Z. contributed equally to this work.

Supporting Information contains:

Supplementary Methods S1-S6

Supplementary Notes S1-S9

Figs. S1 to S16

Tables S1-S3

Contents

Supplementary Methods 3

S1. Chemicals 3

S2. SiO_2 Substrate Preparation 3

S3. hBN Sample Preparation 3

S4. HD-SFG Measurement 3

S5. Suspended Graphene Sample Preparation 4

S6. Machine Learning Potential 4

Supplementary Note 8

S1. Cleanness of the Prepared hBN Surface 8

S2. Screening of Substrate Effect 8

S3. Phase Measurement and Fresnel Factor 9

S4. Effect of Carbonate from CO_2 Dissociation 12

S5. Determination of Surface Charge Density 12

S6. Defect characterization of hBN 14

S7. Stability of the Chemisorbed and Physisorbed States 16

S8. Spectroscopic Evidence for OH^- Chemisorption 20

S9. Fluence-independent SFG Signal 20

References 22

Supplementary Methods

S1. Chemicals

All related chemicals of sodium chloride (NaCl), heavy water (D_2O), sodium hydroxide (NaOH), hydrochloride (HCl, 37%), concentrated sulfuric acid (H_2SO_4, 98%), 30 wt. % hydrogen peroxide solution (H_2O_2), ethanol, and acetone were purchased from Sigma-Aldrich and were all of analytical grade without further purification. Polydimethylsiloxane (PDMS) was provided by Dow, Inc. Deionized water was provided by a Milli-Q system (resistivity ≥ 18.2 MΩ·cm and TOC ≤ 4 ppb). CVD-grown graphene on copper foils was purchased from Grolltex Inc. hBN crystals were obtained from International Center for Materials Nanoarchitectonics, National Institute for Materials Science 1-1 Namiki, Tsukuba 305-0044, Japan.

S2. SiO_2 Substrate Preparation

Water-free SiO_2 substrates (10×10 ×1 mm^3, PI-KEM Ltd) were cleaned with acetone and 2-propanol sequentially. Prior to the transfer of hBN flakes, the SiO_2 substrate was subjected to an oxygen plasma treatment (300 W, 20 sccm O_2, and a duration of 10 minutes). This oxygen plasma treatment was to ensure surface cleanliness and enhance the adhesion with the hBN flakes during the transfer process.

S3. hBN Sample Preparation

High-quality hBN flakes were exfoliated via mechanical cleavage using polydimethylsiloxane (PDMS) substrate (SYLGARD™ 184 Silicone Elastomer Kit, mixed at a 9:1 ratio of base to curing agent). To thin the hBN flakes, we repeatedly exfoliated the flakes using fresh PDMS substrates until the thickness was reduced to less than 100 nm. The final hBN flakes with larger than 200 × 200 μm^2 area were then identified using an optical microscope and dry-transferred onto an oxygen plasma-treated SiO_2 substrate. After flake preparation, a flat and clean region of approximately 150 × 150 μm^2 in size was identified and protected using an optical microscope and shadow mask. Then, a Cr/Au (3nm/100nm) film was deposited on the hBN crystals by electron gun evaporation to mark the identified area for the HD-SFG measurement and cover the edge of the flake and substrate regions. The preparation of the suspended graphene on the water surface was similar to Refs.[1,2] and was detailed in our recent work[3].

S4. HD-SFG Measurement

HD-SFG measurements were performed on a non-collinear beam geometry with a Ti:Sapphire regenerative amplifier laser system. A detailed description can be found in Ref.[4,5]. The measurements

were performed at the *ssp* polarization combination, where *ssp* denotes *s*-polarized SFG, *s*-polarized visible, and *p*-polarized IR beams. The power of the IR and visible beams was approximately 1 mW and 2 mW for measuring the hBN/water interface. We ensured that the IR and visible beam irradiations were far below the damage threshold of hBN (Supplementary Note S8). The IR, visible, and LO beams are directed at the sample (in SiO_2) at incidence angles of approximately 34°, 43°, and 41°, respectively. Each spectrum was acquired with an exposure time of 10 minutes and measured more than 6 times on average. All the HD-SFG spectra were measured in a dried air atmosphere to avoid spectral distortion due to water vapor. To obtain the phase information, the hBN/H_2O HD-SFG signal at *ssp* polarization was normalized with the signal of hBN/D_2O at *ssp* polarization at the same sample spot.

A description of our sample cell can be found in refs[4,5], and will not be elaborated here. For the ion concentration- and pH-dependent HD-SFG measurements, the flow cell was connected to a syringe pump for the supply of solutions. For each measurement, the cell was pumped with the salt solution for ~10 minutes and the processes were repeated three times before the HD-SFG measurement to avoid the memory effect.

S5. Suspended Graphene Sample Preparation

The preparation of the suspended graphene on the water surface was similar to Refs.[1,2] and was detailed in our recent work[3]. In brief, we used large-area monolayer graphene grown on copper foil via chemical vapor deposition (CVD, Grolltex Inc.). For sample preparation, the copper foil was spin-coated with cellulose acetate butyrate (CAB, 30 mg/mL in ethyl acetate) at 1,000 rpm for 10 seconds and 4,000 rpm for 60 seconds, followed by baking at 180 °C for 3 minutes. After cooling to room temperature, the sample was immersed in a $HCl/H_2O_2/H_2O$ solution (volume ratio 1:1:10) for 60 seconds to remove graphene from the backside of the foil. The sample was then rinsed with deionized water, and the CAB layer was dissolved by immersion in acetone. To etch away the copper substrate, the foil was soaked in a 10 mM ammonium persulfate (APS) solution for over 12 hours. The solution was subsequently rinsed and exchanged multiple times with pure water to remove residual etchants.

S6. Machine Learning Potential

Model Development. To develop the MLP, we utilized training data from previous studies[6,7], which included datasets for the hBN-water interface and bulk water under various conditions. In addition, we incorporated configurations specifically targeting the chemisorbed and physisorbed states, as well as

sampling the OH^- ion in water layers farther from the hBN interface. To avoid the need for a homogeneous background charge, we included an H_3O^+ ion positioned far from its counterion to maintain charge neutrality—an approach previously shown to be effective[8,9]. This method eliminates dependencies on the simulation box volume, ensuring the robustness of our MLP. Moreover, it enables the model to accurately describe the behavior of the H_3O^+ ion. To further refine the MLP, we expanded our dataset to include pure water and neutral water containing protonic defects across various environments, including the air-water interface, the hBN-water interface with varying water layer thicknesses, and water confined between hBN sheets. Finally, we performed an additional round of active learning to optimize the model under these diverse conditions.

Electronic Structure Settings. The MLP was developed (and validated) using the energies and forces from the training data obtained at the DFT level. For this, we used the CP2K/Quickstep code[10]. We specifically used the revPBE-D3[11,12] functional as it accurately reproduces the structure and dynamics of liquid water[13–15] and its ionized products[8]. Atomic cores were represented using dual-space GTH pseudopotentials[16]. The Kohn-Sham orbitals of oxygen and hydrogen atoms were expanded using the TZV2P basis set, while the DZVP basis set was used for boron and nitrogen atoms[17]. Additionally, an auxiliary plane-wave basis with a cutoff of 1050 Ry was employed to represent the electron density.

Model Validation. We validated the model's ability to reproduce the reference method by quantifying the root-mean-square error (RMSE) of energies and forces using structures obtained from 250 ps MLP-based MD simulations. These simulations targeted the chemisorbed and physisorbed states.

To evaluate the accuracy and consistency of our results, we performed additional single-point DFT calculations on 800 randomly selected snapshots extracted from the MD simulations, computing their energies and forces. This conforms the additional set of test data to evaluate the MLP developed in this work. To reduce computational costs involved in these DFT single-point calculations, we scaled down the system dimensions, setting the hBN lattice parameters to 13.047 Å × 12.555 Å as opposed to 17.396 Å × 17.577 Å. Unlike the MLP simulations, these configurations included only the OH^- ion without a counterion, necessitating the application of a homogeneous background charge to maintain charge neutrality. To account for the energy shift introduced by this charge when comparing the energies to the MLP, we subtracted a constant energy offset from the MLP-predicted energies. Notably, this validation approach is particularly robust, as it directly assesses structures sampled from the MLP's potential energy

surface, ensuring an accurate comparison with the reference method. As shown in Fig. S1 and Fig. S2, the MLP exhibits strong agreement with the reference DFT calculations for both the chemisorbed and physisorbed states, demonstrating its ability to accurately reproduce the underlying level of theory.

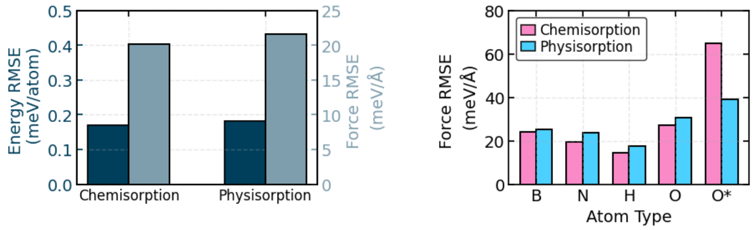

Fig. S1 | RMSE of energies and forces predicted by the MLP compared to reference DFT calculations. The force RMSE is further broken down by atom type, where O* denotes the oxygen atom in the OH^- ion.

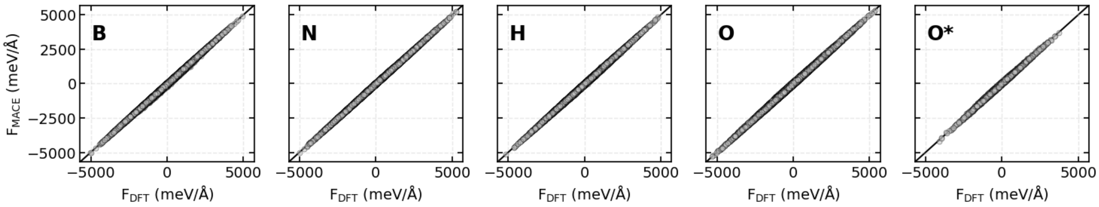

Fig. S2 | Parity plots for the forces obtained using the MLP compared to reference DFT calculations broken down by atom type, where O* denotes the oxygen atom in the OH^- ion.

S1. Molecular Dynamics Simulations

All MD simulations were performed using the MLP at a temperature of 300 K under the NVT ensemble, with a time step of 0.5 fs. Simulations were conducted in orthorhombic cells with periodic boundary conditions applied in all three directions. The systems with no strain were modeled using a 17.396 Å × 17.577 Å × 35.000 Å orthorhombic cell, containing 112 surface atoms, one OH^- ion, and 169 water molecules under periodic boundary conditions. For the strained systems, we applied a ±2% variation along the *x*-axis, resulting in cell dimensions ranging from 17.048 Å × 17.577 Å × 35.000 Å to 17.744 Å × 17.577 Å × 35.000 Å. A representative snapshot of the simulated systems is shown in Fig. S3.

For the free MD simulations, thermalization was achieved using a Langevin thermostat with a friction coefficient of 2.5 ps^{-1}. Each simulation included a 50 ps equilibration phase followed by its corresponding production run.

For the constrained MD simulations used in restrained umbrella sampling, thermalization was achieved using a Nosé-Hoover thermostat with a damping constant of 0.05 ps. A total of 33 umbrella

windows were sampled, each undergoing a 45 ps equilibration phase followed by a 75 ps production run. The reported PMF profile was obtained using umbrella integration.

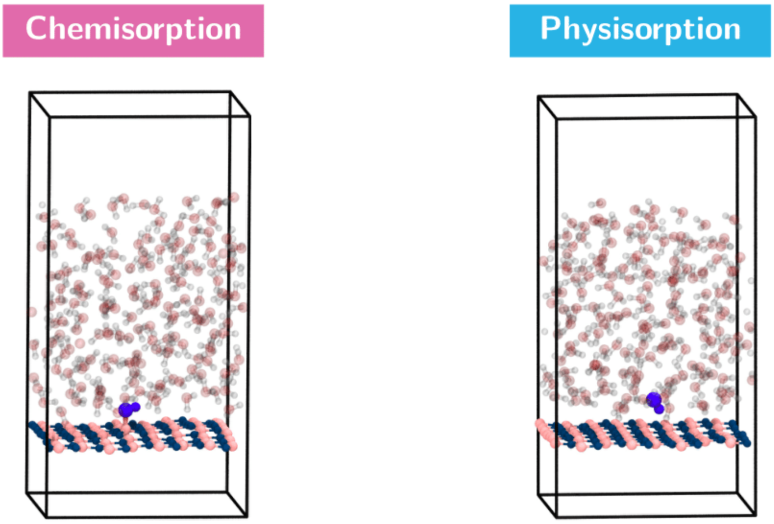

Fig. S3 | Representative snapshot illustrating the dimensions of the systems studied.

In each umbrella window, the oxygen atom of the OH^- ion (O*) was restrained at different target heights above a fixed B atom, while the rest of the hBN interface remained fully flexible. The restraining potential applied took the form:

$$U_{bias,1}(z) = \frac{k_{bias,1}}{2}(z - z_0)^2, \tag{S1}$$

where z is the instantaneous height of the O^* above the hBN sheet, defined as the distance between O and the fixed B atom. The force constant is set to $k_{bias,1} =$ 150 kcal/mol/Å. To avoid proton hopping, we restrained the hydrogen coordination value of the O* around a target value n_0 (here, this is 1.0) using a harmonic potential of the form,

$$U_{bias,2}(z) = \frac{k_{bias,2}}{2}(n_{O*-H} - n_0)^2\,, \tag{S2}$$

where $k_{bias,2} =$ 400 kcal/mol per coordination unit squared and

$$n_{O*-H} = \sum_{i=1}^{N} \frac{1 - \left(\frac{r_i}{R_0}\right)^{12}}{1 - \left(\frac{r_i}{R_0}\right)^{20}}\,, \tag{S3}$$

where i iterates over all the hydrogens in the simulation box, r_i is the distance between a hydrogen i and O*, and R_0 is a switching distance (1.2 Å).

Supplementary Note

S1. Cleanness of the Prepared hBN Surface

To confirm the hBN surface is clean and flat within the HD-SFG probe region (the diameter of the laser spot is around 100 μm), we conducted AFM measurements on the hBN surface across a 100×100 μm^2 region. The large-area surface morphology of the hBN samples was measured using an atomic force microscope (AFM, Bruker, JPK) working in the noncontact mode. We used a silicon cantilever (OPUS-240AC, f = 70 kHz, k = 2 Nm^{-1}) for the measurement. The AFM data shows that the hBN surface appears clean and atomically flat with no visible layered step edges within the SFG probed region, showing an RMS surface roughness (R_q) measuring around 0.7 Å (Fig. S4).

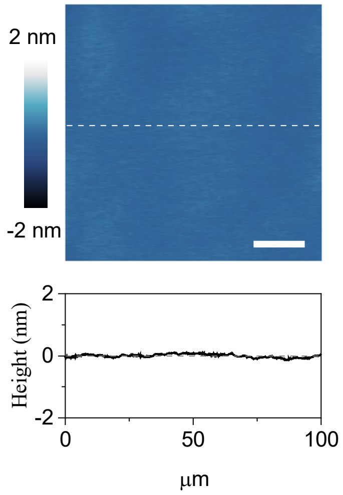

Fig. S4 | Characterization of the hBN surface. AFM height image of the hBN surface. The scale bar is 20 μm. The bottom panel shows the height profiles along the white dashed lines in the AFM height image. The dashed grey lines in the height profiles indicate zero lines. R_q values were calculated across the whole scan area.

S2. Screening of Substrate Effect

The supporting substrate may influence interfacial water arrangement at the substrate-supported two-dimensional materials/water interface, such as substrate-supported monolayer graphene[4,5,18–20]. To avoid the substrate effect, we prepared approximately 100 nm thick hBN flakes. To confirm that the substrate effect is efficiently screened by the approximately 100 nm thick hBN flake, we prepared the hBN flakes using different substrates (SiO_2 and CaF_2) with different polarities and measured the $\mathrm{Im}(\chi^{(2)}_{\mathrm{BN}})$ spectra. The data shown in Fig. S5 confirms that the substrate effect is effectively screened by the approximately 100 nm thick hBN flake.

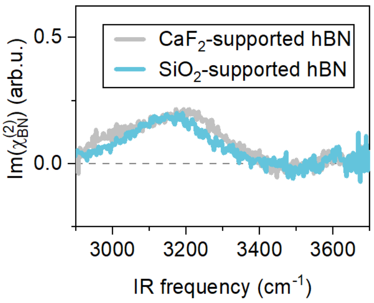

Fig. S5 | Screening of substrate effect. Experimental $\mathrm{Im}\left(\chi^{(2)}_{\mathrm{BN}}\right)$ spectra of pure water obtained at CaF_2- and SiO_2-supported hBN/water interfaces. The grey dashed line represents a zero line.

S3. Phase Measurement and Fresnel Factor

Phase Measurement. To obtain the phase information, the HD-SFG signal ($\chi^{(2)}_{\mathrm{eff},ssp,\mathrm{SiO_2-hBN/H_2O}}$) of the hBN/$H_2O$ interface at *ssp* polarization was normalized by the signal ($\chi^{(2)}_{\mathrm{eff},ssp,\mathrm{SiO_2-hBN/D_2O}}$) of hBN/$D_2O$ interface at *ssp* polarization. The measured SFG response ($\chi^{(2)}_{ssp,\mathrm{measured}}$) is thus given by:

$$\chi^{(2)}_{ssp,\mathrm{measured}} = \frac{\chi^{(2)}_{\mathrm{eff},ssp,\mathrm{SiO_2-hBN/H_2O}}}{\chi^{(2)}_{\mathrm{eff},ssp,\mathrm{SiO_2-hBN/D_2O}}}. \tag{S4}$$

Fresnel Factor Correction. While bulk hBN is SFG-inactive, its surface can exhibit significant non-resonant second-order susceptibility ($\chi^{(2)}_{yyz,\mathrm{hBN}}$), primarily arising from the outermost hBN layer where inversion symmetry is broken. This response is purely real[21–23]. Although both hBN and D_2O response are pure real, for the thin film interface, the Fresnel factor may influence both the amplitude and phase of $\chi^{(2)}_{ssp,\mathrm{measured}}$. To account for these effects, a Fresnel factor correction was conducted. The SiO_2-supported hBN/water interface consists of three bulk media, represented by refractive indices n_1, n_2, and n_3, and two interfacial regions, represented by refractive indices n' and n'', as illustrated in Fig. S6. In such a three-phase system, the effective SFG response ($\chi^{(2)}_{\mathrm{eff},ssp}$) is expressed as[24]:

$$\chi^{(2)}_{\mathrm{eff},ssp} = F^{12}\left(\chi^{(2)}_{yyz,\mathrm{hBN}} + \chi^{(2)}_{yyz,\mathrm{EQ}}\right) + F^{23}\left(-\chi^{(2)}_{yyz,\mathrm{hBN}} - \chi^{(2)}_{yyz,\mathrm{EQ}}\right) + F^{23}\chi^{(2)}_{yyz}, \tag{S5}$$

where F^{12}, F^{23} are Fresnel factors for the SiO_2/hBN ($z = 0$) and hBN/water ($z = d$) interfaces respectively. $\chi^{(2)}_{yyz,\mathrm{EQ}}$ accounts for the non-resonant response at the two interfaces, primarily originating from the electric quadrupole contribution[25,26] which is purely real with its amplitude not highly sensitive

to the refractive indices of the two bulk media forming the interface. We assumed that the amplitudes of $\chi^{(2)}_{yyz,\mathrm{hBN}}$ and $\chi^{(2)}_{yyz,\mathrm{EQ}}$ remain the same for the SiO_2/hBN ($z = 0$) and hBN/water ($z = d$) interfaces, but with opposite sign.

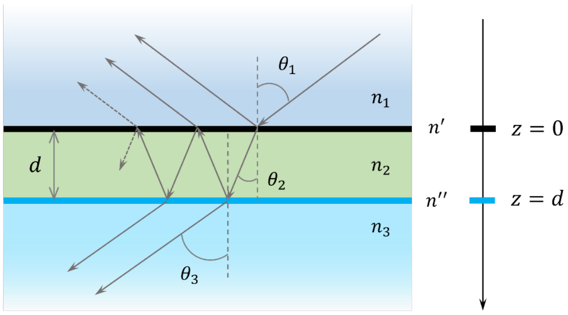

Fig. S6 | Schematic diagram illustrating multiple reflections of an incident light in the hBN film.

Since the non-resonant background ($\chi^{(2)}_{yyz,\mathrm{NR}} = \chi^{(2)}_{yyz,\mathrm{hBN}} + \chi^{(2)}_{yyz,\mathrm{EQ}}$) is explicitly included in Eq. S5, $\chi^{(2)}_{yyz}$ represents the *yyz* component of the resonant SFG signal for H_2O, which is absent for D_2O. As such, from Eq. S4 and S5, the $\chi^{(2)}_{yyz}$ signal at the SiO_2-supported hBN/water interface can be expressed as:

$$\chi^{(2)}_{yyz} = \left(\chi^{(2)}_{ssp,\mathrm{measured}} - \frac{F^{12}_{\mathrm{H_2O}} - F^{23}_{\mathrm{H_2O}}}{F^{12}_{\mathrm{D_2O}} - F^{23}_{\mathrm{D_2O}}}\right)\frac{\left(F^{12}_{\mathrm{D_2O}} - F^{23}_{\mathrm{D_2O}}\right)\chi^{(2)}_{yyz,\mathrm{NR}}}{F^{23}_{\mathrm{H_2O}}}. \tag{S6}$$

The Fresnel factors F^{12} and F^{23} were calculated via[24]:

$$F^{12} = \left(1 + \tilde{r}_{s,12}(\omega_{\mathrm{SF}})\right) \times \left(1 + \tilde{r}_{s,12}(\omega_{\mathrm{vis}})\right) \times \left(1 + \tilde{r}_{p,12}(\omega_{\mathrm{IR}})\right)\left(\frac{n_1}{n'}\right)^2 \sin\theta_1\,, \tag{S7}$$

$$F^{23} = \tilde{t}_{s,23}(\omega_{\mathrm{SF}}) \times \tilde{t}_{s,23}(\omega_{\mathrm{vis}}) \times \tilde{t}_{p,23}(\omega_{\mathrm{IR}})\frac{n_1 n_3}{(n'')^2}\sin\theta_1\,, \tag{S8}$$

where $\tilde{r}$ and $\tilde{t}$ denote the overall reflection and transmission coefficients at the SiO_2-supported hBN/water interface, incorporating multiple reflections within the thin hBN film[24]. They are calculated using Eq. S9 and S10[24]. ω_{SF}, ω_{vis}, and ω_{IR} denote the frequencies of the SF, visible, and IR light, respectively. θ denotes the incident angle. The subscripts *s* and *p* indicate the *s*-polarized and *p*-polarized light, respectively. The indices 1, 2, and 3 correspond to the respective media. Additionally, n' and n'' represent the two interfacial dielectric constants. In this work, the Slab model is employed to describe the two interfacial dielectric constants (n' and n'') which is a commonly used approach for complex multilayer systems under the ssp polarization combination[24].

$$\tilde{r} = r_{12} + \frac{t_{12} r_{23} t_{21} e^{i\Delta\phi}}{1 - r_{21} r_{23} e^{i\Delta\phi}}, \tag{S9}$$

$$\tilde{t} = \frac{t_{12} t_{23} e^{i\Delta\phi/2}}{1 - r_{23} r_{21} e^{i\Delta\phi}}, \tag{S10}$$

where r and t are the Fresnel reflection coefficient and Fresnel transmission coefficient at single interface. Notably, Eq. S9 is used to obtain $\tilde{r}_s$ and $\tilde{r}_p$ depending on whether r_s and t_s or r_p and t_p are utilized. This also applies to Eq. S10. $\Delta\phi = 4\pi d/\lambda\, n_2 \cos\theta_2$ is the propagation phase shift for light (λ, wavelength in a vacuum) passing through the thin hBN layer of thickness $d = 100$ nm. Notably, the thickness of the hBN flake was specifically chosen to ensure that the SFG primarily probes the hBN/water ($z = d$) interface, where F^{23} dominates over F^{12}.

Table S1. Refractive indexes used to calculate the Fresnel factors.

Refractive index *n*	SF (~635 nm)	Vis (800 nm)	IR (3000 nm)
hBN	2.13	2.10	2.00
SiO_2	1.46	1.45	1.41
D_2O	1.33	1.33	1.25

To get $\chi^{(2)}_{yyz}$ via Eq. S6, knowledge of the non-resonant background ($\chi^{(2)}_{yyz,\mathrm{NR}} = \chi^{(2)}_{yyz,\mathrm{hBN}} + \chi^{(2)}_{yyz,\mathrm{EQ}}$) is required. Previous studies have shown that $\chi^{(2)}_{yyz,\mathrm{hBN}}$ is pure real[27], with its amplitude ranging from 0 to 1.5×10^{-20} $\mathrm{m^2/V}$, depending on the thickness and crystal orientation around the *z*-axis of the hBN[21–23]. In our SFG measurements, the hBN crystal orientation around the *z*-axis was manually optimized to be close to the maximum intensity of the homodyne SFG signal at the SiO_2-supported hBN/D_2O interface. This approach enhanced the hBN response, improving overall SFG signal stability and minimizing laser instability effects. Nevertheless, the exact amplitude of $\chi^{(2)}_{yyz,\mathrm{hBN}}$ remains unknown, we instead inferred $\chi^{(2)\prime}_{yyz} = \chi^{(2)}_{yyz}/\chi^{(2)}_{yyz,\mathrm{NR}}$ from Eq. S6. Using the parameters listed in Table S1 and Eq. S6-S10, the inferred $\chi^{(2)\prime}_{yyz}$ is shown in Fig. S7. Notably, for H_2O, frequency-dependent refractive index was employed[28,29]. The results confirm that the Fresnel factors do not alter the main conclusion: the hBN surface is negatively charged upon contacting water.

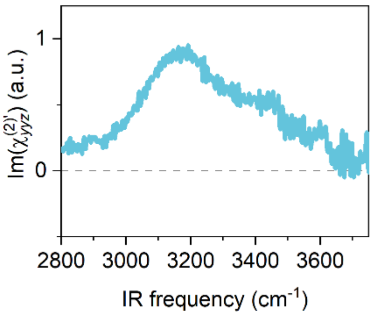

Fig. S7 | Effect of Fresnel Factor. Inferred $\mathrm{Im}\left(\chi^{(2)\prime}_{yyz}\right)$ spectrum of pure water. The grey dashed line represents a zero line.

S4. Effect of Carbonate from CO_2 Dissociation

To examine the effect of carbonate due to CO_2 dissociation in water, we measured the $\mathrm{Im}(\chi^{(2)}_{\mathrm{BN}})$ spectrum using Ar-purged pure water. The data shown in Fig. S8 confirm that carbonate is not responsible for the negative charging of the hBN surface upon contacting water.

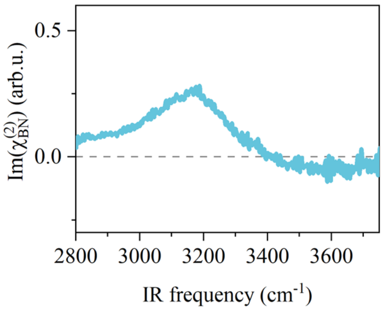

Fig. S8 | Effect of carbonate. Experimental $\mathrm{Im}\left(\chi^{(2)}_{\mathrm{BN}}\right)$ spectrum of Ar-purged pure water. The grey dashed line represents a zero line.

S5. Determination of Surface Charge Density

As discussed in the main text, at charged interfaces, observed $\chi^{(2)}$ is given as the sum of the surface contribution ($\chi^{(2)}_{\mathrm{SL}}$-term) and the DC field-induced bulk contribution ($\chi^{(2)}_{\mathrm{DL}}$-term) within the Gouy-Chapman-Stern (GCS) model[5,30–36]:

$$\chi^{(2)}(c) = \chi^{(2)}_{\mathrm{SL}} + \chi^{(3)}\phi_{\mathrm{s}}(c)\frac{\kappa(c)}{\kappa(c) - i\Delta k_z}, \tag{S11}$$

where ϕ_s is the interfacial electrostatic potential at the plane (z_s) that separates surface and bulk contributions, $\chi^{(3)}$ primarily represents the third-order nonlinear susceptibility originating from bulk water, c is the ion strength, κ is the inverse of Debye screening length, and $\Delta k_z \approx 1/25$ nm^{-1} (@3300 cm^{-1}) is the phase-mismatch of the SF, visible, and IR beams in the depth direction.

Assuming that the $\chi_{\rm SL}^{(2)}$-term is insensitive to the ion strength[30,37] and ions (≲100 mM NaCl) do not affect the hBN surface charging, the $\chi^{(2)}$ spectral changes upon ion strength changes primarily arise from the variation of the $\chi_{\rm DL}^{(2)}$-term at the charged interface. This allows for determination of the $\chi^{(3)}$ spectrum from the differential spectrum. To this end, we measured the $\chi_{\rm BN}^{(2)}$ spectra at three different ion strengths at neutral pH~6. We used the ion strengths of $c_1 = 1$ µM, $c_2 = 10$ mM, and $c_3 = 100$ mM (see Fig. S9a). Combined with We then obtained $\sigma_{\rm s}$ from the differential spectra $\Delta\chi_{\rm BN}^{(2)}(\sigma_{\rm s}, c_i, c_j) = \chi_{\rm BN}^{(2)}(\sigma_{\rm s}, c_i) - \chi_{\rm BN}^{(2)}(\sigma_{\rm s}, c_j)$ via[38]:

$$\frac{\Delta\chi^{(2)}(\sigma_{\rm s}, c_1, c_3)}{\Delta\chi^{(2)}(\sigma_{\rm s}, c_2, c_3)} = \frac{\left(\frac{\phi_{\rm s}(\sigma_{\rm s}, c_1)\kappa(c_1)}{\kappa(c_1) - i\Delta k_z} - \frac{\phi_{\rm s}(\sigma_{\rm s}, c_3)\kappa(c_3)}{\kappa(c_3) - i\Delta k_z}\right)}{\left(\frac{\phi_{\rm s}(\sigma_{\rm s}, c_2)\kappa(c_2)}{\kappa(c_2) - i\Delta k_z} - \frac{\phi_{\rm s}(\sigma_{\rm s}, c_3)\kappa(c_3)}{\kappa(c_3) - i\Delta k_z}\right)}. \quad \text{(S12)}$$

With known $\sigma_{\rm s}$, the $\chi^{(3)}$ spectrum was obtained via:

$$\Delta\chi^{(2)}(\sigma_{\rm s}, c_2, c_3) = \chi^{(3)}\left(\frac{\phi(\sigma_{\rm s}, c_2)\kappa(c_2)}{(\kappa(c_2) - i\Delta k_z)} - \frac{\phi(\sigma_{\rm s}, c_3)\kappa(c_3)}{(\kappa(c_3) - i\Delta k_z)}\right). \quad \text{(S13)}$$

The obtained $\chi^{(3)}$ spectrum is shown in Fig. S9b. The lineshape of the spectrum is consistent with that reported in Refs.[30]. Once $\chi^{(3)}$ is known, Eq. S13 allows us to estimate $\sigma_{\rm s}$ at different pH values. The differential spectra $\mathrm{Im}(\Delta\chi_{\rm BN}^{(2)})$ at different pH values are presented in Fig. S9c and corresponding inferred $\sigma_{\rm s}$ are shown in Fig. 3b.

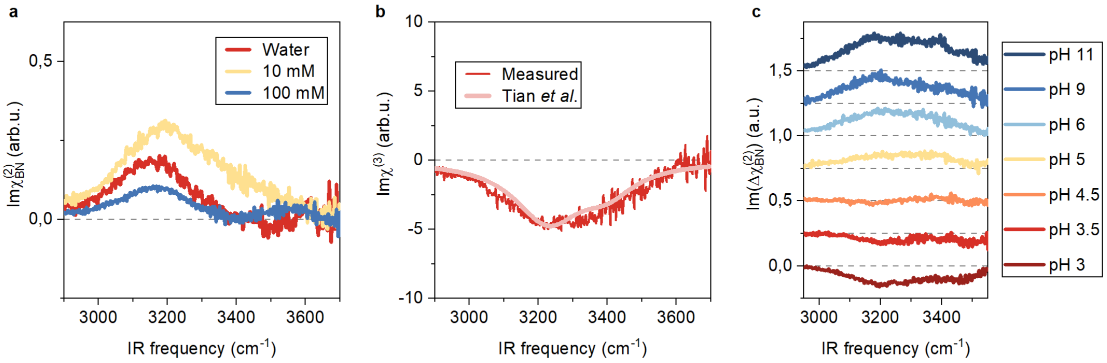

Fig. S9 | Measurement of $\chi^{(3)}$ spectrum. a. Experimental $\mathrm{Im}\left(\chi_{\rm BN}^{(2)}\right)$ spectra at different ion strengths at pH~6. The dashed line represents the zero line. **b**. Comparison of the measured $\mathrm{Im}(\chi^{(3)})$ spectrum and that reported in Ref.[30]. Note that $\mathrm{Im}(\chi^{(3)})$ spectrum from Ref.[30] is rescaled in a way that the peak amplitude at ~3250 cm^{-1} has the same value as that in our data. **c**. Experimental $\mathrm{Im}\left(\Delta\chi_{\rm BN}^{(2)}\right)$ spectra at different pH values obtained from $\Delta\chi_{\rm BN}^{(2)} = \chi_{\rm BN}^{(2)}(\sigma_{\rm s}, c_2 = 10\ \mathrm{mM}) - \chi_{\rm BN}^{(2)}(\sigma_{\rm s}, c_3 = 100\ \mathrm{mM})$. The grey dashed lines in (**a-c**) represent zero lines.

We note that the method used to estimate σ_s relies on the Gouy-Chapman-Stern model[5,30–32], which assumes that the $\chi^{(2)}_{SL}$-term is insensitive to ion strength and that ions (≤100 mM NaCl) do not influence the charging of the hBN surface. To examine the ion concentration effect, we also inferred σ_s from $\Delta\chi^{(2)}_{BN}$ obtained from the SFG signal of 1 mM and 100 mM NaCl. The $\mathrm{Im}(\Delta\chi^{(2)}_{BN})$ data is shown in Fig. S10 and inferred σ_s is -10 mC/m^2, slightly smaller than that inferred from difference spectrum $\Delta\chi^{(2)}_{BN}$ between 10 mM and 100 mM NaCl solutions. This analysis indicates that the NaCl ion concentration influences the surface charging of hBN; however, it does not alter our main conclusion that hBN undergoes spontaneous negative surface charging upon contact with water.

Accurately estimating σ_s requires further refinement of the Gouy-Chapman-Stern model to account for both surface and bulk contributions in the SFG signal, such as ion-induced surface discharging[39,40] and a more precise determination of the position of z_s, which remains a hot topic at the current stage[41]. This level of detail is beyond the scope of the present study and warrants additional investigation in future work.

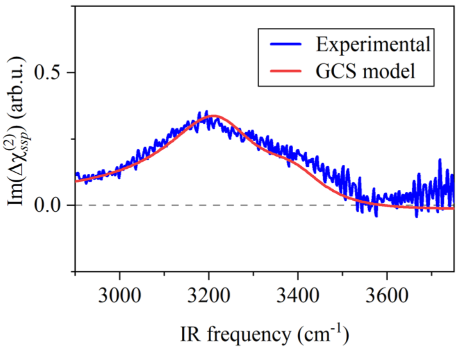

Fig. S10 | Influence of ions on hBN surface charging. Experimental difference spectrum $\mathrm{Im}\left(\Delta\chi^{(2)}_{BN}\right)$ between 1 mM and 100 mM NaCl solutions, compared with a calculated spectrum based on the Gouy-Chapman-Stern theory for σ_s =-10 mC/m^2. The grey dashed line represents a zero line.

S6. Defect characterization of hBN

To ensure the absence of defects on the hBN surface, we conducted qPlus-based AFM measurements over different randomly selected regions. Consistent with the data shown in Fig. 1f-h, the constant-height, high-resolution AFM images of the hBN surface from another randomly selected region reveal a clean surface with a perfect hexagonal honeycomb structure without any defects over an area of 100 nm² (Fig. S11).

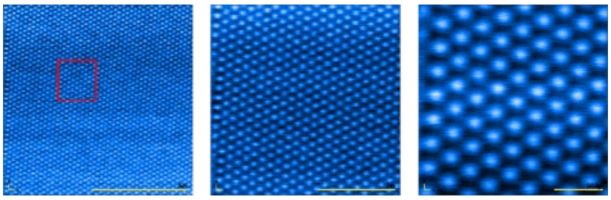

Fig. S11 | qPlus-based AFM characterization of hBN. a. Constant-height AFM image of the hBN surface. **b** and **c**. Zoomed-in AFM images from (**a**) with B and N atoms marked. The scale bars are 5 nm, 2 nm, and 0.5 nm, respectively.

Based on the surface charge estimated from SFG data at the hBN/water interface, which is approximately -15 mC/m^2, this corresponds to approximately one charge per 10.67 nm^2. Assuming a Poisson distribution of surface defects, the probability of finding no defect within an area of 100 nm^2 is given by Eq. S14:

$$P_0 = e^{-\lambda}, \tag{S14}$$

where $\lambda = 100\ \mathrm{nm}^2/10.67\ \mathrm{nm}^2$ is the expected number of surface charges/defects in that area. From five independent AFM measurements, the combined probability of finding no defects in all measurements is:

$$P = P_0^5 \approx 4.4 \times 10^{-21}. \tag{S15}$$

To further assess whether water contact or SFG measurements induce observable defects on an initially defect-free hBN surface, we performed Raman spectroscopy on freshly exfoliated hBN on a SiO_2 substrate (fresh hBN) and compared it with spectrum obtained after ~2 hours of SFG laser irradiation in contact with water (exposed hBN). Measurements were taken from the same topmost surface, and each spectrum was averaged over five different sample spots—under air for the fresh sample and under water for the exposed sample. In both cases, the spectra exhibit a prominent E_{2g} band at 1367 cm^{-1} and no detectable defect-related D_1 band (~1290 cm^{-1})[42], indicating no laser- or water-induced defect formation (Fig. S12). The E_{2g} band amplitude is reduced for the exposed hBN, which we attribute to attenuation of both excitation and emission signals by ~100 μm of water and a 170 μm glass window in this measurement. Notably, the Raman spectrum of the same hBN sample after drying (dried hBN) shows negligible change in amplitude. These results confirm that neither water exposure nor SFG laser irradiation induces observable defects on the hBN surface.

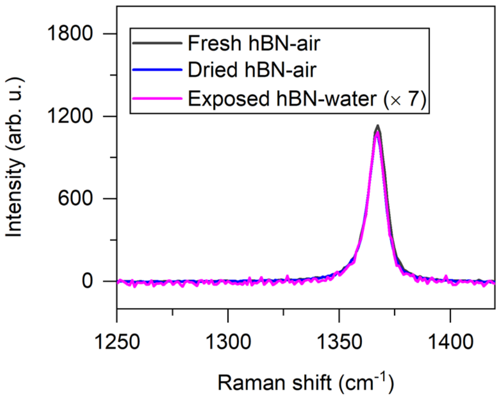

Fig. S12 | Raman characterization of the hBN sample. Raman spectra of freshly exfoliated hBN, after exposure to water and SFG laser irradiation, and after drying. The Raman spectrum of the exposed hBN was magnified 7× for comparison. All Raman spectra were recorded with a WITec confocal Raman spectrometer (Alpha 300 R, ×50 objective) with 1200 grooves/mm grating, 532 nm laser, 3 mW power, and 30 s integration time.

S7. Stability of the Chemisorbed and Physisorbed States

To gain deeper insights into the stability of the chemisorbed and physisorbed states, we conducted additional free MD simulations, tracking the desorption time from the chemisorbed state (Table S2).

To further quantify the low free energy barrier between these states, we estimated the activation energy (E_a) using an Arrhenius-like expression:

$$E_a = k_B T \ln(A\tau) \,, \tag{S16}$$

where E_a is the activation energy, k_B is the Boltzmann constant, T is the temperature, A is the pre-exponential factor (assumed to be 10^{13} s^{-1}), and τ is the desorption time obtained from our simulations. This estimation provides a direct connection between the observed timescales and the energetic barriers governing the transition between adsorption states.

Table S2. Free MD simulations starting from the chemisorbed state.

Run	Time for chemisorbed to physisorbed transition (ps)	Estimated activation energy, E_a (eV)
#1	85	0.17

#2	370	0.21
#3	80	0.17
#4	1685	0.25
#5	750	0.23
#6	460	0.22
#7	1270	0.24

As shown in Table S2, the estimated barriers align well with the barriers obtained from the PMF in Fig. 2a, further validating our approach. Similarly, we can also provide an estimate of the time required by an OH^- ion to transition between the physisorbed and chemisorbed states. This can be done by rearranging Eq. S16 for τ, where E_a is obtained from the PMF in Fig. 2a. The data is shown in Table S3.

Table S3. Estimated times required by an OH^- ion to transition between the states.

	E_a	τ
Physisorbed to chemisorbed	0.36 eV	111.62 ns
Chemisorbed to physisorbed	0.25 eV	1.58 ns

The comparable stability of the chemisorbed and physisorbed states suggests that nuclear quantum effects (NQEs) or exchange-correlation (XC) functional dependency may play a crucial role.

In the case of NQEs, their primary contribution can be approximated through the zero-point energy (ZPE), which, to a first-order approximation, is given by:

$$\mathrm{ZPE} = \frac{1}{2}\hbar\omega, \tag{S17}$$

where ω represents the vibrational frequency. This frequency can be estimated using:

$$\omega = \sqrt{\frac{k}{m}}, \tag{S18}$$

where k is the force constant, which can be determined by fitting a harmonic potential to the potential of mean force (PMF) in Fig. 2a, as done above. Substituting appropriately in Eq. S17, we calculate the ZPE for the chemisorbed state to be 0.0931 eV, while for the physisorbed state, it is 0.0275 eV. This difference highlights the steeper free energy well and higher vibrational frequency of the chemisorbed state compared to the physisorbed state. This is an admittedly crude semi-quantitative estimate of NQEs, neglecting for example the zero point energy of the other vibrational models in the system as well as any potential anharmonic effects. However, the magnitude of the ZPE difference between the two states implies that their relatively stability is unlikely to be greatly affected by NQEs.

The DFT XC functional is always an important consideration with simulating aqueous systems[13]. We have chosen the revPBE-D3 functional here as it accurately reproduces the structure and dynamics of liquid water[13–15] and its ionized products[8]. However, revPBE-D3 is a generalized gradient approximation (GGA) functional and it is well known that GGA functionals can overestimate electrostatic contributions to binding energies[43] and introduce delocalization errors[44]. These problems are largely ameliorated with hybrid functionals in which a fraction of exact (Hartree-Fock) exchange is introduced. To investigate this issue, we compared the system's total energies with the OH^- ion in either the chemisorbed or the physisorbed state, using the revPBE-D3 and hybrid revPBE0-D3 functionals. For this analysis, we selected 300 configurations for the chemisorbed state and 300 configurations for the physisorbed state from MD simulations.

As shown in Fig. S13, both the revPBE-D3 and hybrid revPBE0-D3 functionals predict similar total energies between the physisorbed and chemisorbed states. In particular, revPBE0-D3 shifts the stability toward the chemisorbed state by 0.039 eV, making it more favorable. This effect is opposite to the influence of NQEs, which instead slightly stabilized the physisorbed state. As a result, these two contributions cancel each other to some extent, further highlighting the competitive balance between these states and reinforcing that these factors do not alter the main conclusions of our work.

Lastly, to further explore the differences between the chemisorbed and physisorbed states, we

examined the diffusive behavior of the OH^- ion in both configurations, as shown in Fig. S14. Our analysis reveals clear differences in their dynamics. In the chemisorbed state, OH^- remains largely fixed to the boron atom it is bonded to, exhibiting minimal movement. In contrast, in the physisorbed state, OH^- shows in-plane mobility, allowing it to diffuse more freely along the surface. This difference in mobility is particularly relevant for nanoscale friction on hBN.

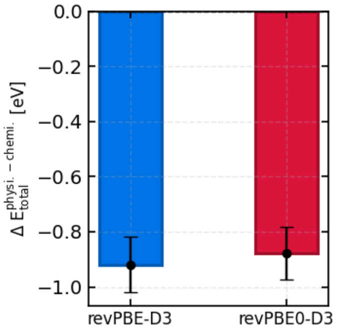

Fig. S13 | Difference in the total energy of the system when the OH^- ion is in either the chemisorbed or the physisorbed state obtained with the revPBE-D3 functional (the functional the MLP used in the main text is trained on) and the hybrid revPBE0-D3 functional. The total energy of the chemisorbed state serves as the reference (set to zero). Error bars represent the standard deviation from 300 sampled configurations for each state.

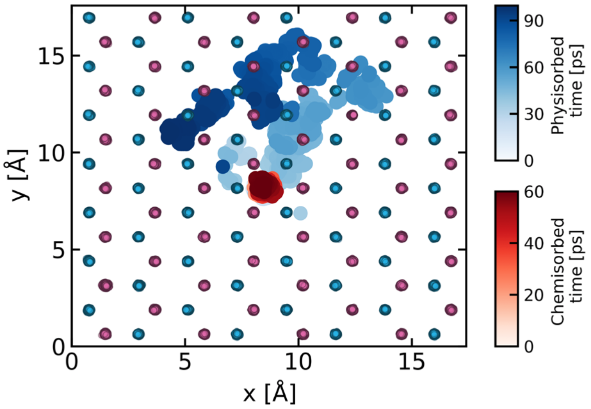

Fig. S14 | In-plane motion of the OH^- ion in the chemisorbed and physisorbed states. The positions are represented by the oxygen of the hydroxide ion. The color gradient represents the time in picoseconds for each state. In this simulation, the OH^- started in the chemisorbed state and transitioned to the physisorbed state at approx. 60 ps.

S8. Spectroscopic Evidence for OH^- Chemisorption

The SFG signal at neutral pH do not show the signature of chemisorbed OH group (expected a negative high-frequency peak around 3600–3670 cm^{-1}), probably because of the low abundance of OH^- at pH ~6. Indeed, our HD-SFG spectra measured at pH 11 show a weak negative peak at ~3620 cm^{-1}, as presented in Fig. 3a. To further confirm the reliability of this weak feature, we measured the HD-SFG signal at pH 11 with an improved signal-to-noise ratio. The resulting data shown in Fig. S15 clearly reveal a negative peak at ~3620 cm^{-1}. The high frequency of this peak indicates a non-hydrogen-bonded O–H stretch, while its negative sign suggests that the O–H group is oriented down, toward the bulk solution. We thus assign this feature to the stretch vibrational mode of a chemisorbed OH group on the hBN surface.

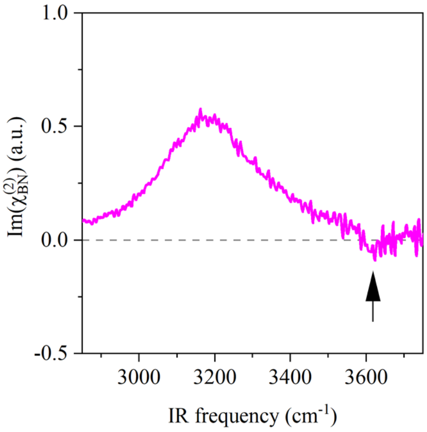

Fig. S15 | Chemisorbed OH group revealed by HD-SFG spectroscopy. Experimental $\mathrm{Im}\left(\chi^{(2)}_{\mathrm{BN}}\right)$ spectra obtained for 100 mM NaCl at pH 11. The black arrow indicates the 3620 cm^{-1} peak. The grey dashed line serves as a zero line.

S9. Fluence-independent SFG Signal

To ensure the usage of 1 mW for IR (~3.3 μm) and 2 mW visible (800 nm) pulses do not damage the hBN sample, we compared the $\mathrm{Im}(\chi^{(2)}_{\mathrm{BN}})$ signals measured at different fluences. If the IR and visible pulses do damage the hBN sample, more defects (charges) are expected upon increasing the pulse power. The data displayed in Fig. S16 shows the water arrangement remains the same within the experimental uncertainty by increasing the power of IR and visible pulses, showing the IR and visible pulses do not introduce defects on the hBN surface.

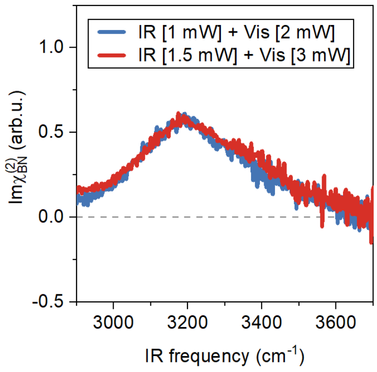

Fig. S16 | Stability of hBN in contact with water under laser irradiation. Experimental $\mathrm{Im}\left(\chi^{(2)}_{\mathrm{BN}}\right)$ spectra obtained for water (10 mM NaCl) at pH~6 under different IR/vis fluences.

References

(1) Xu, Y.; Ma, Y.-B.; Gu, F.; Yang, S.-S.; Tian, C.-S. Structure Evolution at the Gate-Tunable Suspended Graphene–Water Interface. *Nature* **2023**, *621* (7979), 506–510. https://doi.org/10.1038/s41586-023-06374-0.

(2) Yang, S.; Zhao, X.; Lu, Y.-H.; Barnard, E. S.; Yang, P.; Baskin, A.; Lawson, J. W.; Prendergast, D.; Salmeron, M. Nature of the Electrical Double Layer on Suspended Graphene Electrodes. *J. Am. Chem. Soc.* **2022**, *144* (29), 13327–13333. https://doi.org/10.1021/jacs.2c03344.

(3) Wang, Y.; Tang, F.; Yu, X.; Ohto, T.; Nagata, Y.; Bonn, M. Heterodyne-Detected Sum-Frequency Generation Vibrational Spectroscopy Reveals Aqueous Molecular Structure at the Suspended Graphene/Water Interface. *Angew. Chem. Int. Ed.* **2024**, *63* (20), e202319503. https://doi.org/10.1002/anie.202319503.

(4) Wang, Y.; Seki, T.; Yu, X.; Yu, C.-C.; Chiang, K.-Y.; Domke, K. F.; Hunger, J.; Chen, Y.; Nagata, Y.; Bonn, M. Chemistry Governs Water Organization at a Graphene Electrode. *Nature* **2023**, *615* (7950), E1–E2. https://doi.org/10.1038/s41586-022-05669-y.

(5) Wang, Y.; Seki, T.; Liu, X.; Yu, X.; Yu, C.-C.; Domke, K. F.; Hunger, J.; Koper, M. T. M.; Chen, Y.; Nagata, Y.; Bonn, M. Direct Probe of Electrochemical Pseudocapacitive pH Jump at a Graphene Electrode**. *Angew. Chem. Int. Ed.* **2023**, *62* (10), e202216604. https://doi.org/10.1002/anie.202216604.

(6) Thiemann, F. L.; Schran, C.; Rowe, P.; Müller, E. A.; Michaelides, A. Water Flow in Single-Wall Nanotubes: Oxygen Makes It Slip, Hydrogen Makes It Stick. *ACS Nano* **2022**, *16* (7), 10775–10782. https://doi.org/10.1021/acsnano.2c02784.

(7) Ravindra, P.; Advincula, X. R.; Schran, C.; Michaelides, A.; Kapil, V. Quasi-One-Dimensional Hydrogen Bonding in Nanoconfined Ice. *Nat. Commun.* **2024**, *15* (1), 7301. https://doi.org/10.1038/s41467-024-51124-z.

(8) Atsango, A. O.; Morawietz, T.; Marsalek, O.; Markland, T. E. Developing Machine-Learned Potentials to Simultaneously Capture the Dynamics of Excess Protons and Hydroxide Ions in Classical and Path Integral Simulations. *J. Chem. Phys.* **2023**, *159* (7), 074101. https://doi.org/10.1063/5.0162066.

(9) Advincula, X. R.; Fong, K. D.; Michaelides, A.; Schran, C. Protons Accumulate at the Graphene-Water Interface. arXiv February 4, 2025. https://doi.org/10.48550/arXiv.2408.04487.

(10) Kühne, T. D.; Iannuzzi, M.; Del Ben, M.; Rybkin, V. V.; Seewald, P.; Stein, F.; Laino, T.; Khaliullin, R. Z.; Schütt, O.; Schiffmann, F.; Golze, D.; Wilhelm, J.; Chulkov, S.; Bani-Hashemian, M. H.; Weber, V.; Borštnik, U.; Taillefumier, M.; Jakobovits, A. S.; Lazzaro, A.; Pabst, H.; Müller, T.; Schade, R.; Guidon, M.; Andermatt, S.; Holmberg, N.; Schenter, G. K.; Hehn, A.; Bussy, A.; Belleflamme, F.; Tabacchi, G.; Glöß, A.; Lass, M.; Bethune, I.; Mundy, C. J.; Plessl, C.; Watkins, M.; VandeVondele, J.; Krack, M.; Hutter, J. CP2K: An Electronic Structure and Molecular Dynamics Software Package - Quickstep: Efficient and Accurate Electronic Structure Calculations. *J. Chem. Phys.* **2020**, *152* (19), 194103. https://doi.org/10.1063/5.0007045.

(11) Perdew, J. P.; Burke, K.; Ernzerhof, M. Generalized Gradient Approximation Made Simple. *Phys.*

Rev. Lett. **1996**, *77* (18), 3865–3868. https://doi.org/10.1103/PhysRevLett.77.3865.

(12) Grimme, S.; Antony, J.; Ehrlich, S.; Krieg, H. A Consistent and Accurate Ab Initio Parametrization of Density Functional Dispersion Correction (DFT-D) for the 94 Elements H-Pu. *J. Chem. Phys.* **2010**, *132* (15), 154104. https://doi.org/10.1063/1.3382344.

(13) Gillan, M. J.; Alfè, D.; Michaelides, A. Perspective: How Good Is DFT for Water? *J. Chem. Phys.* **2016**, *144* (13), 130901. https://doi.org/10.1063/1.4944633.

(14) Morawietz, T.; Singraber, A.; Dellago, C.; Behler, J. How van Der Waals Interactions Determine the Unique Properties of Water. *Proc. Natl. Acad. Sci. U.S.A.* **2016**, *113* (30), 8368–8373. https://doi.org/10.1073/pnas.1602375113.

(15) Marsalek, O.; Markland, T. E. Quantum Dynamics and Spectroscopy of Ab Initio Liquid Water: The Interplay of Nuclear and Electronic Quantum Effects. *J. Phys. Chem. Lett.* **2017**, *8* (7), 1545–1551. https://doi.org/10.1021/acs.jpclett.7b00391.

(16) Goedecker, S.; Teter, M.; Hutter, J. Separable Dual-Space Gaussian Pseudopotentials. *Phys. Rev. B* **1996**, *54* (3), 1703–1710. https://doi.org/10.1103/PhysRevB.54.1703.

(17) VandeVondele, J.; Hutter, J. Gaussian Basis Sets for Accurate Calculations on Molecular Systems in Gas and Condensed Phases. *J. Chem. Phys.* **2007**, *127* (11), 114105. https://doi.org/10.1063/1.2770708.

(18) Kim, D.; Kim, E.; Park, S.; Kim, S.; Min, B. K.; Yoon, H. J.; Kwak, K.; Cho, M. Wettability of Graphene and Interfacial Water Structure. *Chem* **2021**, *7* (6), 1602–1614. https://doi.org/10.1016/j.chempr.2021.03.006.

(19) Montenegro, A.; Dutta, C.; Mammetkuliev, M.; Shi, H.; Hou, B.; Bhattacharyya, D.; Zhao, B.; Cronin, S. B.; Benderskii, A. V. Asymmetric Response of Interfacial Water to Applied Electric Fields. *Nature* **2021**, *594* (7861), 62–65. https://doi.org/10.1038/s41586-021-03504-4.

(20) Wang, Y.; Nagata, Y.; Bonn, M. Substrate Effect on Charging of Electrified Graphene/Water Interfaces. *Faraday Discuss.* **2023**. https://doi.org/10.1039/D3FD00107E.

(21) Bernhardt, N.; Kim, S.; Fröch, J. E.; White, S. J. U.; Duong, N. M. H.; He, Z.; Chen, B.; Liu, J.; Aharonovich, I.; Solntsev, A. S. Large Few-Layer Hexagonal Boron Nitride Flakes for Nonlinear Optics. *Opt. Lett., OL* **2021**, *46* (3), 564–567. https://doi.org/10.1364/OL.416564.

(22) Kim, S.; Fröch, J. E.; Gardner, A.; Li, C.; Aharonovich, I.; Solntsev, A. S. Second-Harmonic Generation in Multilayer Hexagonal Boron Nitride Flakes. *Opt. Lett., OL* **2019**, *44* (23), 5792–5795. https://doi.org/10.1364/OL.44.005792.

(23) Li, Y.; Rao, Y.; Mak, K. F.; You, Y.; Wang, S.; Dean, C. R.; Heinz, T. F. Probing Symmetry Properties of Few-Layer MoS2 and h-BN by Optical Second-Harmonic Generation. *Nano Lett.* **2013**, *13* (7), 3329–3333. https://doi.org/10.1021/nl401561r.

(24) Moloney, E. G.; Azam, Md. S.; Cai, C.; Hore, D. K. Vibrational Sum Frequency Spectroscopy of Thin Film Interfaces. *Biointerphases* **2022**, *17* (5), 051202. https://doi.org/10.1116/6.0002085.

(25) Yamaguchi, S. (山口祥一); Shiratori, K. (白鳥和矢); Morita, A. (森田明弘); Tahara, T. (田原太

平). Electric Quadrupole Contribution to the Nonresonant Background of Sum Frequency Generation at Air/Liquid Interfaces. *J. Chem. Phys.* **2011**, *134* (18), 184705. https://doi.org/10.1063/1.3586811.

(26) Shen, Y. R. Revisiting the Basic Theory of Sum-Frequency Generation. *J. Chem. Phys.* **2020**, *153* (18), 180901. https://doi.org/10.1063/5.0030947.

(27) Vandelli, M.; Katsnelson, M. I.; Stepanov, E. A. Resonant Optical Second Harmonic Generation in Graphene-Based Heterostructures. *Phys. Rev. B* **2019**, *99* (16), 165432. https://doi.org/10.1103/PhysRevB.99.165432.

(28) Yu, X.; Chiang, K.-Y.; Yu, C.-C.; Bonn, M.; Nagata, Y. On the Fresnel Factor Correction of Sum-Frequency Generation Spectra of Interfacial Water. *J. Chem. Phys.* **2023**, *158* (4), 044701. https://doi.org/10.1063/5.0133428.

(29) Hale, G. M.; Querry, M. R. Optical Constants of Water in the 200-Nm to 200-Mm Wavelength Region. *Appl. Opt., AO* **1973**, *12* (3), 555–563. https://doi.org/10.1364/AO.12.000555.

(30) Wen, Y.-C.; Zha, S.; Liu, X.; Yang, S.; Guo, P.; Shi, G.; Fang, H.; Shen, Y. R.; Tian, C. Unveiling Microscopic Structures of Charged Water Interfaces by Surface-Specific Vibrational Spectroscopy. *Phys. Rev. Lett.* **2016**, *116* (1), 016101. https://doi.org/10.1103/PhysRevLett.116.016101.

(31) Ohno, P. E.; Wang, H.; Geiger, F. M. Second-Order Spectral Lineshapes from Charged Interfaces. *Nat. Commun.* **2017**, *8* (1), 1032. https://doi.org/10.1038/s41467-017-01088-0.

(32) Reddy, S. K.; Thiraux, R.; Wellen Rudd, B. A.; Lin, L.; Adel, T.; Joutsuka, T.; Geiger, F. M.; Allen, H. C.; Morita, A.; Paesani, F. Bulk Contributions Modulate the Sum-Frequency Generation Spectra of Water on Model Sea-Spray Aerosols. *Chem* **2018**, *4* (7), 1629–1644. https://doi.org/10.1016/j.chempr.2018.04.007.

(33) Fellows, A. P.; Duque, Á. D.; Balos, V.; Lehmann, L.; Netz, R. R.; Wolf, M.; Thämer, M. Sum-Frequency Generation Spectroscopy of Aqueous Interfaces: The Role of Depth and Its Impact on Spectral Interpretation. *J. Phys. Chem. C* **2024**, *128* (49), 20733–20750. https://doi.org/10.1021/acs.jpcc.4c06650.

(34) Wei, F.; Urashima, S.; Nihonyanagi, S.; Tahara, T. Elucidation of the pH-Dependent Electric Double Layer Structure at the Silica/Water Interface Using Heterodyne-Detected Vibrational Sum Frequency Generation Spectroscopy. *J. Am. Chem. Soc.* **2023**, *145* (16), 8833–8846. https://doi.org/10.1021/jacs.2c11344.

(35) Hsiao, Y.; Chou, T.-H.; Patra, A.; Wen, Y.-C. Momentum-Dependent Sum-Frequency Vibrational Spectroscopy of Bonded Interface Layer at Charged Water Interfaces. *Sci. Adv.* **2023**, *9* (15), eadg2823. https://doi.org/10.1126/sciadv.adg2823.

(36) Tetteh, N.; Parshotam, S.; Gibbs, J. M. Separating Hofmeister Trends in Stern and Diffuse Layers at a Charged Interface. *J. Phys. Chem. Lett.* **2024**, *15* (35), 9113–9121. https://doi.org/10.1021/acs.jpclett.4c01792.

(37) Joutsuka, T.; Morita, A. Electrolyte and Temperature Effects on Third-Order Susceptibility in Sum-Frequency Generation Spectroscopy of Aqueous Salt Solutions. *J. Phys. Chem. C* **2018**, *122* (21),

11407–11413. https://doi.org/10.1021/acs.jpcc.8b02445.

(38) Seki, T.; Yu, X.; Zhang, P.; Yu, C.-C.; Liu, K.; Gunkel, L.; Dong, R.; Nagata, Y.; Feng, X.; Bonn, M. Real-Time Study of on-Water Chemistry: Surfactant Monolayer-Assisted Growth of a Crystalline Quasi-2D Polymer. *Chem* **2021**, *7* (10), 2758–2770. https://doi.org/10.1016/j.chempr.2021.07.016.

(39) Li, Z.; Hall, A. T.; Wang, Y.; Li, Y.; Byrne, D. O.; Scammell, L. R.; Whitney, R. R.; Allen, F. I.; Cumings, J.; Noy, A. Ion Transport and Ultra-Efficient Osmotic Power Generation in Boron Nitride Nanotube Porins. *Sci. Adv.* **2024**, *10* (36), eado8081. https://doi.org/10.1126/sciadv.ado8081.

(40) Siria, A.; Poncharal, P.; Biance, A.-L.; Fulcrand, R.; Blase, X.; Purcell, S. T.; Bocquet, L. Giant Osmotic Energy Conversion Measured in a Single Transmembrane Boron Nitride Nanotube. *Nature* **2013**, *494* (7438), 455–458. https://doi.org/10.1038/nature11876.

(41) R. Advincula, X.; G. Backus, E. H.; Bonn, M.; J. Cox, S.; Diebold, U.; Fellows, A.; R. Finney, A.; Goel, G.; Hedley, J.; Jiang, Y.; Jin, D.; Kapil, V.; Kavokine, N.; Klein, J.; Laage, D.; Mohandas, N.; Morgenstern, K.; Mukherjee, T.; Cruz, M. O. de la; Orlikowska-Rzeznik, H.; Perkin, S.; M. Piaggi, P.; Gomez Rodellar, C.; Ryan, P.; Sayer, T.; Seyffertitz, M.; Shepelenko, M.; C. Sosso, G.; Thämer, M.; Vilangottunjalil, A.; Walker-Gibbons, R.; Wang, Y.; P. Willard, A.; Zhang, P. Electrified/Charged Aqueous Interfaces: General Discussion. *Faraday Discuss.* **2024**, *249* (0), 381–407. https://doi.org/10.1039/D3FD90065G.

(42) Patra, A.; Konrad, P.; Sperlich, A.; Biktagirov, T.; Schmidt, W. G.; Spencer, L.; Aharonovich, I.; Höfling, S.; Dyakonov, V. Quantifying Spin Defect Density in hBN via Raman and Photoluminescence Analysis. arXiv June 24, 2025. https://doi.org/10.48550/arXiv.2506.19803.

(43) Otero-de-la-Roza, A.; Johnson, E. R. Analysis of Density-Functional Errors for Noncovalent Interactions between Charged Molecules. *J. Phys. Chem. A* **2019**. https://doi.org/10.1021/acs.jpca.9b10257.

(44) Bryenton, K. R.; Adeleke, A. A.; Dale, S. G.; Johnson, E. R. Delocalization Error: The Greatest Outstanding Challenge in Density-Functional Theory. *WIREs Comput. Mol. Sci.* **2023**, *13* (2), e1631. https://doi.org/10.1002/wcms.1631.
